# Exploring Collagen Parameters in Pure Special Types of Invasive Breast Cancer

**DOI:** 10.1038/s41598-019-44156-9

**Published:** 2019-05-22

**Authors:** Rodrigo de Andrade Natal, Geisilene R. Paiva, Vitor B. Pelegati, Ludwing Marenco, César A. Alvarenga, Renato F. Vargas, Sophie F. Derchain, Luis O. Sarian, Camille Franchet, Carlos L. Cesar, Fernando C. Schmitt, Britta Weigelt, José Vassallo

**Affiliations:** 1 Laboratory of Investigative and Molecular Pathology, CIPED – Faculty of Medical Sciences – State University of Campinas, Rua Tessália Vieira de Camargo, 126, Zip code: 13083-970, Campinas, São Paulo Brazil; 2 Laboratory of Specialized Pathology, LAPE – Faculty of Medical Sciences – State University of Campinas, Rua Tessália Vieira de Camargo, 126, Zip code: 13083-970, Campinas, São Paulo Brazil; 3 Department of Quantum Electronics –Institute of Physics “Gleb Wataghin” – State University of Campinas, Rua Sérgio Buarque de Holanda, 777, Zip code: 13083-859, Campinas, São Paulo Brazil; 4 Instituto de Patologia de Campinas (Private Laboratory), Av. Andrade Neves, 1801, Zip Code: 13070-000, Campinas, São Paulo Brazil; 5 Department of Obstetrics and Gynecology – Faculty of Medical Sciences – State University of Campinas, Rua Tessália Vieira de Camargo, 126, Zip code: 13083-970, Campinas, São Paulo Brazil; 6 Department of Pathology, University Cancer Institute, Avenue Irene Joliot Curie, 1, Zip code: 31059, Toulousse, France; 7 Department of Physics, Federal University of Ceará (UFC), Campus do Pici - Bloco 922 - Zip code: 60455-760, Fortaleza, Ceará Brazil; 8 Institute of Molecular Pathology and Immunology of Porto University (IPATIMUP) – Porto University, Rua Dr. Roberto Frias, s/n, Zip code: 4200-465, Porto, Portugal; 9 National Santé Laboratory, Department of Medicine – L-3555, Dudelange, Luxembourg; 10 Department of Pathology – Memorial Sloan Kettering Cancer Center, York Avenue 1275, Zip code: 10065, New York, USA

**Keywords:** Biological fluorescence, Oncogenesis, Nonlinear optics

## Abstract

One of the promising tools to evaluate collagen in the extracellular matrix is the second-harmonic generation microscopy (SHG). This approach may shed light on the biological behavior of cancers and their taxonomy, but has not yet been applied to characterize collagen fibers in cases diagnosed as invasive breast carcinoma (BC) of histological special types (IBC-ST). Tissue sections from 99 patients with IBC-ST and 21 of invasive breast carcinoma of no special type (IBC-NST) were submitted to evaluation of collagen parameters by SHG. Tissue microarray was performed to evaluate immunohistochemical-based molecular subtype. In intratumoral areas, fSHG and bSHG (forward-SHG and backward-SHG) collagen parameters achieved their lowest values in mucinous, papillary and medullary carcinomas, whereas the highest values were found in classic invasive lobular and tubular carcinomas. Unsupervised hierarchical cluster analysis and minimal spanning tree using intratumoral collagen parameters allowed the identification of three main groups of breast cancer: group A (classic invasive lobular and tubular carcinomas); group B (IBC-NST, metaplastic, invasive apocrine and micropapillary carcinomas); and group C (medullary, mucinous and papillary carcinomas). Our findings provide further characterization of the tumor microenvironment of IBC-ST. This understanding may add information to build more consistent tumor categorization and to refine prognostication.

## Introduction

Invasive breast carcinoma is considered a combination of heterogeneous diseases, encompassing multiple entities with distinct biological and clinical features. The most common subtype of breast carcinoma is invasive ductal carcinoma of no special type (IBC-NST), formerly known as invasive ductal carcinoma, which accounts for around 50% to 80% of all breast cancers^1–3^. The remaining tumor subtypes are collectively referred to as invasive carcinoma with histological special types (IBC-ST), and account for approximately 25% of invasive breast carcinomas^1–4^. Although pathologists have been aware of the diversity of breast cancers and have endeavored to devise approaches to classify the disease into meaningful groups^3,5–9^, this concept has only been brought to the forefront of breast cancer research after the publication of high-throughput microarray-based discovery studies that unraveled the existence of multiple molecular subtypes^10–13^.

IBC-ST refers to the architectural growth of the tumors, defining special morphological and cytological patterns, which have been consistently associated with distinctive clinical presentation and/or outcomes^3^. However, although histological grade identifies prognostic subgroups in IBC-NST, among the IBC-ST some subtypes show high grade but still bear favorable prognosis (e.g. medullary, mucinous and invasive apocrine breast carcinomas)^3^. Interestingly, genetic and transcriptomic features of breast cancers has been correlated with histological grade, and microarray-based genomic signatures for histological grades have been devised^14–17^.

The use of new investigation tools in IBC-ST has been limited partly due to their relative low prevalence with consequent lower interobserver reproducibility, skewing their systematic investigation in class discovery^10–13^ and class prediction^16,18–21^. This is the case of tumor microenvironment, especially the extracellular matrix (ECM), which has been intensively studied in tumor progression and outcome^22^. Among the various components of ECM, collagen fibers are the most abundant in breast cancer stroma^23,24^. The restructuring of collagen pattern may be either induced by tumor cells, or occur as a result of an integrated modification between neoplastic cell and stroma, suggesting that progression of a tumor relies not only on changes of cell function, but also on ECM alterations.

One of the promising tools to evaluate collagen in the ECM during cancer progression is the second-harmonic generation microscopy (SHG)^25–27^. This approach may shed light on the biological behavior of cancers and their taxonomy^28,29^. It allows for the identification of collagen maturation process by distinguishing collagen diameters. Thus, backward SHG images exhibit punctate distribution attributable to small-diameter, segmental collagen, which permits recognition of fibrillogenesis in immature tissue by directly imaging backward-propagating SHG (bSHG). By contrast, images from the mature fibrils are identical in the forward (fSHG) and backward directions^30^. Although it has not been applied to IBC-ST, several studies have shown that this is an efficient means to describe common breast diseases, differentiate benign from malignant breast tissue and establish prognosis in invasive ductal carcinoma^31–35^. It should be emphasized that studies also indicate that certain aspects of collagen deposition may lead to a more aggressive behavior of the tumor, like greater amount of collagen, presence of perpendicular fibers to the tumor bounder and thicker collagen fibers^34,36–38^. Thus, the goals of the present study are: (1) to investigate peri- and intratumoral collagen parameters in various histological subtypes of pure IBC-ST, using IBC-NST as standard samples, and (2) correlate these parameters with pathological and clinical features.

## Results

### Clinicopathological features

Patients’ age, histological grade, lymph node status and immunohistochemical-based molecular subtype^39^ are shown in Table 1. The median age was 56 years [Interquartile range (IQR) = 45–66 years]. Among all 120 cases, most carcinomas were of histological grade III (n = 48; 40.0%), followed by grade I (n = 43; 35.8%) and grade II (n = 29; 24.2%) carcinomas. The majority of the patients did not present lymph node metastases (n = 54; 45.0%); these were found in 25 patients (20.8%). According to the status of immunohistochemical expression of hormone receptors (HR) and HER2/*neu* protein used as surrogate for molecular types, cases were classified as follows: HR+HER2− was the most frequent subtype (n = 55; 45.8%), followed by HR−HER2− (n = 26; 21.7%), HR+HER2+ (n = 4; 3.3%), and HR−HER2 + (n = 4; 3.3%). In 31 cases (25.8%), patients had no further material for immunohistochemical staining, thus immunohistochemical-based molecular subtyping was not possible for these patients.

**Table 1:** Clinicopathological features of breast carcinoma cases.

Histological types	n (%)	Median (IQR^#^), years	Histological grade			Lymph node status			Molecular subtype				
			I	II	III	Negative	Positive	Not available	HR+ HER2−	HR+ HER2+	HR− HER2+	HR− HER2−	Not available
Classic invasive lobular	7 (07.1%)	57 (57–59)	3	2	2	2	1	4	5	—	—	1	1
Tubular	13 (13.1%)	50 (45–64)	13	—	—	9	2	2	8	—	—	—	5
Mucinous	23 (23.2%)	65 (47–74)	17	5	1	11	4	8	15	—	—	—	8
Mucinous A	13 (13.1%)	66 (54–76)	12	1	—	6	3	4	8	—	—	—	5
Mucinous B	10 (10.1%)	63 (42–70)	5	4	1	5	1	4	7	—	—	—	3
Papillary	9 (09.1%)	61 (52–79)	1	5	3	6	1	2	8	—	—	—	1
Micropapillary	6 (06.1%)	48 (42–63)	—	2	4	1	4	1	4	1	—	—	1
Medullary	15 (15.2%)	47 (38–54)	—	2	13	9	4	2	1	—	—	10	4
Typical	6 (06.1%)	48 (36–58)	—	—	6	4	2	—	—	—	—	6	—
Atypical	9 (09.1%)	47 (45–51)	—	2	7	5	2	2	1	—	—	4	4
Metaplastic	9 (09.1%)	51 (33–56)	—	4	5	2	—	7	—	—	—	8	1
Matrix-producing	4 (04.0%)	56 (50–59)	—	2	2	1	—	3	—	—	—	4	—
Squamous cell	4 (04.0%)	40 (35–46)	—	1	3	1	—	3	—	—	—	3	1
Spindle cell	1 (01.0%)	8 (NA)	—	1	—	—	—	1	—	—	—	1	—
Apocrine	17 (17.1%)	61 (46–69)	—	3	14	8	6	3	4	3	4	3	3
**Total (IBC-ST)**	**99 (100.0%)**	**56 (45–66)**	**43**	**29**	**48**	**54**	**25**	**41**	**45**	**04**	**04**	**22**	**24**
**IBC-NST**	**21**	**58 (50–61)**	**9**	**6**	**6**	**6**	**3**	**12**	**10**	**—**	**—**	**4**	**7**

^#^IQR = interquartile range.

### Collagen features in breast cancer

**Figure 1:**
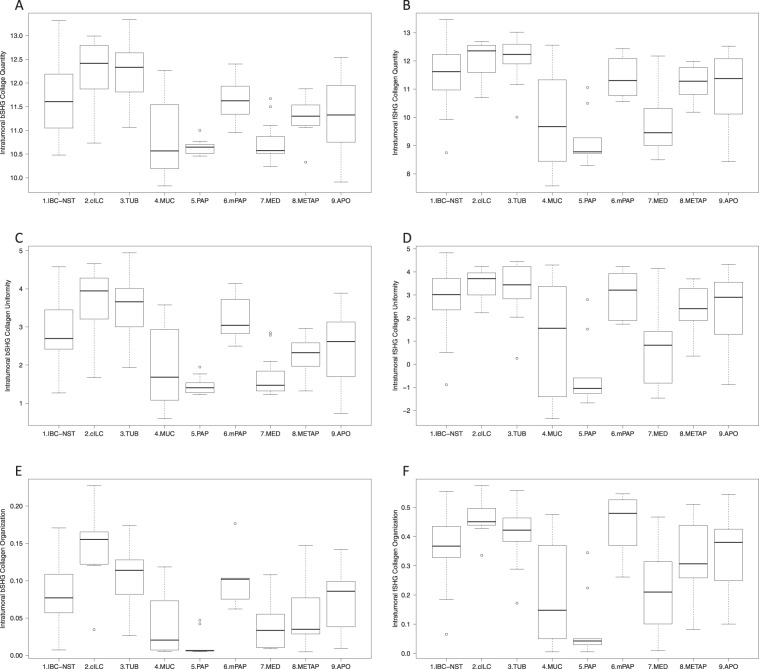
Boxplots demonstrating the distribution of intratumoral collagen parameters in histological subtypes: (**A**) bSHG collagen quantity; (**B**) fSHG collagen quantity; (**C**) bSHG collagen uniformity; (**D**) fSHG collagen uniformity; (**E**) bSHG collagen organization; (**F**) fSHG collagen organization. Classic invasive lobular (2. cILC) and tubular (3. TUB) carcinomas presented the highest values for intratumoral collagen parameters. Mucinous (4. MUC), papillary (5. PAP) and medullary (7. MED) presented the lowest values. Invasive ductal carcinoma of no special type (1. IBC-NST), micropapillary (6. mPAP), metaplastic (8. METAP) and invasive apocrine (9. APO) presented intermediary values of intratumoral collagen parameters. The statistically significant relations are shown in Supplementary Table 3. [bSHG, fSHG: backward and forward propagation second harmonic generation, respectively].

The correlation between collagen parameters (quantity, uniformity and organization) in peritumoral areas was poor, contrasting to high correlation coefficients found in intratumoral areas (Supplementary Table 1 and Fig. 1).

In general, collagen quantity, uniformity and organization were higher in peritumoral regions compared to intratumoral areas (Supplementary Table 2). In classic invasive lobular, tubular, micropapillary and metaplastic carcinomas, there was no statistical difference between fSHG peri- and intratumoral collagen organization. The same was observed for bSHG peri- and intratumoral collagen organization in classic invasive lobular and micropapillary carcinomas.

Peritumoral collagen parameters for both fSHG and bSHG fibers did not differ significantly across histological subtypes (Table 2 and Supplementary Fig. 2). In contrast, in intratumoral areas, fSHG and bSHG collagen quantity, uniformity and organization achieved the lowest values in mucinous, papillary and medullary carcinomas, whereas the highest values for these parameters were found in classic invasive lobular and tubular carcinomas (Table 2, Fig. 1, Supplementary Table 3).

**Table 2:** Collagen parameters in invasive breast carcinoma.

Collagen parameters	IBC-NST	Classic invasive lobular	Tubular	Mucinous	Papillary	Micropapillary	Medullary	Metaplasic	Apocrine
**Peritumoral**									
bSHG collagen quantity^†^	13.61 (0.42)	13.84 (0.46)	13.86 (0.34)	13.58 (0.42)	13.39 (0.31)	13.74 (0.52)	13.34 (0.51)	13.20 (0.47)	13.48 (0.35)
fSHG collagen quantity^†^	14.03 (0.48)	14.19 (0.52)	14.05 (0.35)	13.85 (0.43)	13.75 (0.56)	14.13 (0.52)	13.79 (0.65)	13.75 (0.43)	13.87 (0.30)
bSHG collagen uniformity^†^	5.08 (0.50)	5.40 (0.56)	5.36 (0.39)	5.11 (0.51)	4.81 (0.38)	5.24 (0.56)	4.82 (0.58)	4.64 (0.52)	4.89 (0.42)
fSHG collagen uniformity^†^	5.84 (0.70)	6.07 (0.69)	5.82 (0.47)	5.67 (0.58)	5.37 (0.92)	5.94 (0.58)	5.67 (0.81)	5.59 (0.48)	5.63 (0.49)
bSHG collagen organization^†^	0.16 (0.04)	0.19 (0.04)	0.17 (0.05)	0.15 (0.04)	0.16 (0.05)	0.17 (0.06)	0.16 (0.04)	0.15 (0.03)	0.15 (0.03)
fSHG collagen organization^†^	0.44 (0.07)	0.49 (0.08)	0.48 (0.08)	0.44 (0.08)	0.44 (0.08)	0.49 (0.17)	0.45 (0.05)	0.42 (0.05)	0.45 (0.09)
**Intratumoral**									
bSHG collagen quantity^†^	11.68 (0.73)	12.21 (0.80)	12.31 (0.66)	10.80 (0.76)	10.64 (0.17)	10.64 (0.50)	10.73 (0.41)	11.26 (0.43)	11.30 (0.74)
fSHG collagen quantity^†^	11.56 (1.04)	12.00 (0.74)	12.13 (0.82)	9.83 (1.68)	9.21 (0.95)	11.40 (0.76)	9.83 (1.14)	11.21 (0.62)	11.02 (1.02)
bSHG collagen uniformity^†^	2.86 (0.86)	3.61 (1.02)	3.50 (0.90)	1.93 (1.01)	1.45 (0.25)	3.21 (0.61)	1.67 (0.53)	2.28 (0.53)	2.44 (0.94)
fSHG collagen uniformity^†^	2.86 (1.31)	3.44 (0.74)	3.25 (1.18)	0.99 (2.37)	−0.42 (1.54)	3.04 (1.05)	0.83 (1.73)	2.44 (1.02)	2.35 (1.60)
bSHG collagen organization^†^	0.08 (0.04)	0.14 (0.06)	0.11 (0.04)	0.04 (0.04)	0.01 (0.02)	0.10 (0.04)	0.04 (0.03)	0.05 (0.04)	0.07 (0.04)
fSHG collagen organization^†^	0.37 (0.12)	0.46 (0.08)	0.41 (0.10)	0.18 (0.16)	0.08 (0.12)	0.44 (0.11)	0.21 (0.14)	0.33 (0.13)	0.34 (0.13)

^†^Data in: Mean (Standard deviation).

Peritumoral and intratumoral bSHG collagen quantity (p = 0.008 and p = 0.003, respectively) and uniformity (p = 0.009 and p = 0.005, respectively) were higher in histological grade I tumors compared to grade III tumors. However, there was no difference between peri- and intratumoral collagen quantity and uniformity, considering histological grade II tumors and grade I or histological grade II and grade III tumors. Peri- and intratumoral collagen organization was similar across histological grades (Supplementary Table 4). Peritumoral collagen quantity, uniformity and organization did not differ in relation to lymph node status and immunohistochemical-based subtype. However, intratumoral collagen bSHG fiber uniformity and organization were higher in HR−HER2− tumors compared to HR−HER2+ tumors (Supplementary Table 5).

The multivariate recursive partitioning method, using intratumoral collagen parameters, showed that fSHG and bSHG quantity, uniformity and organization were lower in mucinous, papillary and medullary tumors (p < 0.001). On the other hand, the three parameters were significantly higher in classic invasive lobular and tubular carcinomas (p = 0.005) compared to the other histological subtype. IBC-NST, micropapillary, metaplastic and invasive apocrine carcinomas presented an intermediary fSHG and bSHG intratumoral collagen parameters (Supplementary Fig. 3).

**Figure 2:**
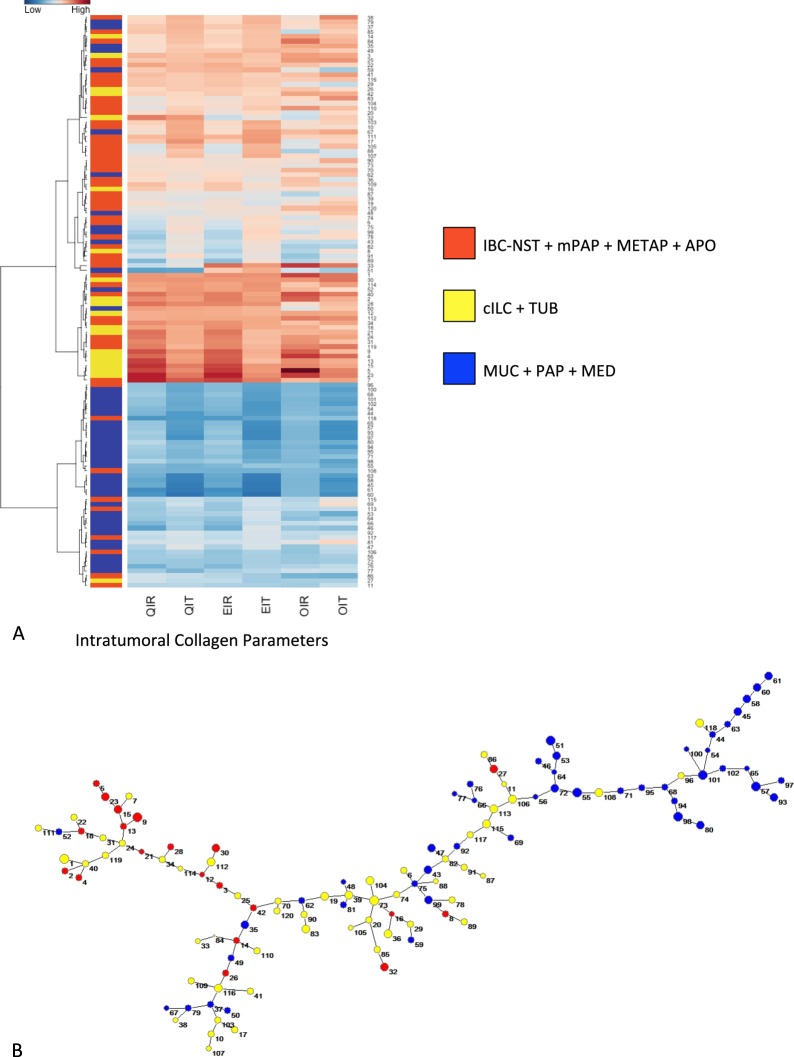
(**A**) Unsupervised hierarchical clustering of intratumoral collagen parameters using Ward algorithms and Euclidian distance; the lowest values for intratumoral collagen parameters refer to mucinous (MUC), papillary (PAP) and medullary (MED) breast carcinomas (light blue); the highest values refer to classical invasive lobular (cILC) and tubular carcinomas (TUB) (light yellow); the intermediary values refer to invasive ductal carcinoma of no special type (IBC-NST), micropapillary (mPAP), metaplastic (METAP) and invasive apocrine (APO) (light orange), being the latter group very heterogeneous. QIR: bSHG collagen quantity; QIT: fSHG collagen quantity; EIR: bSHG collagen uniformity; EIT: fSHG collagen uniformity; OIR: bSHG collagen organization; and OIT: fSHG collagen organization. (**B**) Minimal Spanning Tree (MST) of the Euclidian matrix with three defined intratumoral collagen parameters. The MSP gives a spatial representation of what is shown in the heatplot. Cases presenting similar collagen parameters are linked by a line. As result, cases belonging to the same group (blue, yellow and orange) are concentrated in three branches of the tree: blue in the superior part, yellow in the middle and right region and orange in the left branch.

**Figure 3:**
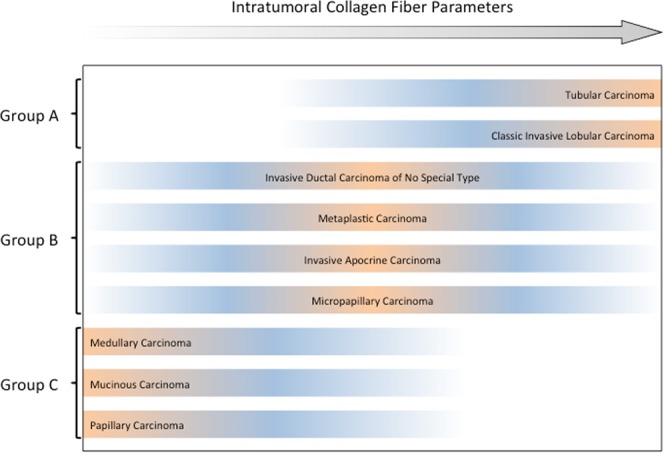
Distribution of breast cancer histological subtypes per intratumoral collagen parameters. Tubular and invasive lobular breast carcinoma showed the highest intratumoral collagen parameters (quantity, uniformity and organization), while medullary, papillary and mucinous breast carcinoma presented the lowest intratumoral collagen parameters. Invasive ductal, metaplastic, invasive apocrine and micropapillary breast carcinomas presented intermediary intratumoral collagen parameters.

Unsupervised hierarchical cluster analysis using intratumoral collagen (Fig. 2 and Supplementary Figs 4 and 5) allowed the identification of three main groups of breast cancer. Group A: high intratumoral collagen quantity, uniformity and organization (classic invasive lobular and tubular carcinomas); group B: intermediary intratumoral collagen quantity, uniformity and organization (invasive ductal, metaplastic, invasive apocrine and micropapillary carcinomas); and group C: low intratumoral collagen quantity, uniformity and organization (medullary, mucinous and papillary carcinomas). Supervised analysis using estrogen receptor (ER) expression or molecular subtype presented the same result. Figure 3 visually summarizes the distinct groups of breast carcinomas according to intratumoral collagen parameters.

### Mucinous, medullary and metaplastic breast carcinoma subclassification

Collagen parameters did not differ between mucinous A and B breast carcinoma. In addition, peritumoral collagen parameters in typical and atypical medullary were similar. Intratumoral fSHG collagen quantity (p = 0.017) and uniformity (p = 0.044) were higher in atypical medullary breast cancer; these findings may be extended to bSHG fiber quantity (p = 0.054) and uniformity (p = 0.054); fSHG and bSHG fiber organization was similar in both tumor subtypes (p = 0.198 and p = 0.064, respectively).

There were no differences in collagen parameters comparing metaplastic breast carcinoma with squamous cells and with matrix-producing breast carcinomas, except for peritumoral fSHG collagen organization, that was higher in metaplastic breast carcinoma with squamous cells (p = 0.017).

## Discussion

Our results allowed grouping breast carcinomas according to the intratumoral collagen parameters (Fig. 3). Group A, characterized by higher intratumoral collagen quantity, uniformity and organization, was enriched for classic invasive lobular and tubular carcinomas; group B, comprised by intermediary intratumoral collagen quantity, uniformity and organization, was enriched for IBC-NST, metaplastic, invasive apocrine and micropapillary carcinomas; and group C, covered lower intratumoral collagen quantity, uniformity and organization, was enriched for medullary, mucinous and papillary carcinomas. Furthermore, it would be worth highlighting that IBC-NST are not only heterogeneous at the morphologic and molecular level, but also at the clustering analysis performed herein; cases of IBC-NST could be found in any of the possible three groups identified, even if we used a supervised clustering analysis.

The findings on intratumoral collagen corroborate the disparities between morphological classification and molecular biology. Heterogeneous tumor biology may be found within the same histological subtype, whereas distinct histological categories share common molecular features^39,40^. As such, morphologic subtyping may be challenging not only because of subjectivity, but also as a result of molecular similarities. Peritumoral collagen fibers presented similar parameters among all histological subtypes of breast cancer, suggesting a common endpoint, shared by them, in the mechanism of collagen deposition at tumor periphery. This acknowledgement may support the pivotal interplay of tumor microenvironment in cancer outcome.

Classic invasive lobular and tubular carcinomas, constituting group A in the present study, have been described as showing remarkably similar immunohistochemical and transcriptomic profiles^39^. Notwithstanding, invasive lobular carcinoma can be differentiated from tubular carcinoma based on the expression levels of E-cadherin, which is absent in the former^41,42^. Regarding collagen parameters, both histological subtypes presented similar intratumoral features, and shared the same hierarchical clustering, supporting the acquaintance between them. These findings provide structural evidence for the hypothesis that classic invasive lobular and tubular carcinomas, both frequently members of the low-grade breast carcinoma group, might originate from the same family of low-grade precursors^41^. Lopez-Garcia *et al*. support the existence of a “low-grade breast neoplasia family”; nevertheless, the transcriptomes of these lesions display small, yet important differences, which together with their distinct biological behavior, warrant their separation as discrete entities^43^.

In opposition to the subtypes above, a clear disparity in the clinical, morphological, molecular or immunohistochemical profiles was found within the other two groups of tumors with similar intratumoral collagen parameters, groups B and C^39,40,44^. This is evidence that, although the microenvironment may importantly contribute to progression of cancer in all phases, factors intrinsic to the neoplastic cells may respond for other important features, as the phenotype and genetic characteristics^39,40^.

Subtypes included in our group B have been described as presenting discrepant clinicopathological behavior. Metaplastic and micropapillary carcinomas present mostly poor outcome, in spite of ER status, respectively, negative and positive; apocrine carcinoma, tipically ER-negative, present variable outcome^45,46^. In a study of genomic profiling, carcinomas with apocrine differentiation, invasive micropapillary and metaplastic carcinomas showed the highest frequencies of chromosome aberrations (i.e. gene amplifications, gains and losses)^40^. Thus, although this group seems heterogeneous as for clinical outcome and for the variable expression of ER, the collagen parameters behaved similarly, and paralleled the high numbers of chromosome aberrations. It may be assumed that the collagen signature of group B is predictive of higher frequency of finding genetic alterations.

On the other hand, group C, enriched for medullary, mucinous and papillary carcinomas have been related to a more indolent clinical behavior^45^. The three subtypes in group C have been described with similar gross growth pattern in image exams, presenting as a round or oval tumor with well-defined contours^47^. Genetic results demonstrated that papillary carcinomas are a homogenous special histological subtype of breast carcinoma, with good prognosis. They tend to present low rates of lymph node metastasis and low p53 expression, ER-positive, low frequency of gene copy number aberrations and high prevalence of PIK3CA mutations. The genomic profile of its three morphological variants (i.e. encapsulated, solid and invasive papillary) has shown remarkably similar^46^. Likewise, mucinous breast carcinoma display ER-positivity, low level of genetic instability and rare recurrent amplifications^48–51^. These genetic features reasonably approximate both tumors in hierarchic clusters^39^. In opposition, medullary carcinomas have been shown to share the basal-like immunophenotype (i.e. HR negative, HER-2 negative and P-cadherin positive), and high frequency of chromosome aberrations^39,40,52–55^. In spite of this divergence in profiling, medullary carcinomas show, in common with the other two members of group C, the favorable prognosis and the lower intratumoral collagen quantity, uniformity and organization. As such, this group is homogeneous only in respect to clinical behavior for all three members^45^.

Metaplastic and medullary carcinomas, grouped herein as B and C, respectively, are consistently of triple-negative phenotype, and both were shown to display similar high frequency of gene copy number aberrations and comparable gene expression patterns^39,40,52–55^. In relation to the concordance between the molecular subtyping and other prognostic gene signatures, patients with the basal-like type should be classified as aggressive tumor^9,56^. However, basal-like medullary carcinomas present a good outcome in opposition to metaplastic carcinoma^3^. Then, in contrast to molecular grouping, intrinsic intratumoral collagen parameters clustering proposed in this study assembled these subtypes of breast carcinomas more suitably.

The rarity of the entities analyzed herein represents a drawback. Additional studies of special subtypes of breast carcinoma will be required to validate these findings, and to determine the biological and clinical relevance of clustering these tumors according to the intratumoral collagen parameters. In special, studies on patients’ outcome for each individual subtype will require larger cohorts. Also, the long collection period might affect the evaluation of collagen structure, although previous studies pointed out that tissue fixation time did not interfere with the availability of macromolecules^57,58^. Further, the present study evaluated images only with the 40×/1.3 oil immersion magnification, largely used in the literature. It would be interesting to specifically address the comparison of collagen parameters values between different magnifications, in order to evaluate the impact of microscopic resolution for these analyses.

Previous studies have already demonstrated the prognostic value of collagen parameters in breast cancer, showing that high disorganization of collagen fibers, i. e. TACS3, is indicative of poorer prognosis^34,37^. In a study on the evaluation of fibrils deposition in collagen fibers, it was also found that different patterns are related to the molecular subtypes^59^. However, most of them did not emphasize the usage of this technique on IBC-ST, with the exception of the study by Conklin *et al*., in which collagen parameters were evaluated in invasive lobular carcinoma^34^. In addition, these studies have not compared different collagen signatures in the histological subtypes. Further, the method applied in the present study was quantitative, in contrast to the qualitative or semiquantitative approach of the previous ones. Besides, the results presented herein could provide the basis for future automated analyses with specific mathematical algorithms for classification, as already described^60^. However, this application is still potential, as, to the best of our knowledge, our study represents the first approach to compare collagen parameters in IBC-ST.

In summary, our results do not allow a prognostic stratification, but provide information that could contribute to refine diagnosis. In that sense, an example would be the differential diagnosis between medullary carcinomas and triple-negative IBC-NST, since these entities belong to different groups, as proposed here. Our results provide a step forward to the characterization of the tumor microenvironment of IBC-ST. This understanding may add information to build more consistent tumor categorization, together with molecular findings. Grouping the different histological subtypes according to collagen parameters, as suggested herein, may represent a plausible explanation for the observation that tumors with different genetic profiles share similar clinical behavior. It potentially contributes to the refinement of the microenvironmental phylomorphology, and of the prognostication of breast cancer patients.

## Materials and Methods

### Case selection

Formalin-fixed, paraffin-embedded (FFPE) tissue specimens from 120 patients with pure invasive breast carcinoma were consecutively retrieved from four centers of Pathology (two in Brazil, one in Portugal and one in France). Tissues were obtained from primary lumpectomy or mastectomy; no patient had undergone primary chemo- or radiotherapy. Tumors were classified based on the WHO criteria^3^: 21 IBC-NST, 7 classic invasive lobular, 13 tubular, 23 mucinous, 6 invasive micropapillary, 9 invasive papillary, 15 medullary, 9 metaplastic and 17 apocrine carcinomas, from January 2000 to January 2015. Mucinous carcinoma were subdivided into hypocellular mucinous (mucinous A; n = 13) and hypercellular mucinous carcinoma (mucinous B; n = 10) based on the criteria of Capella *et al*.^61^. All cases were reviewed on hematoxylin and eosin-stained (H&E) sections by at least two experienced breast pathologists (GRP, CAA, RVF and FCS). The study is fully compliant with the Declaration of Helsinki (approved by the “Comitê de Ética em Pesquisa da Unicamp”).

In order to confirm diagnosis and evaluate immunohistochemical-based molecular subtype^39,44^, two representative intratumoral areas of 2 mm in diameter from each case were selected to build a tissue microarray (tissue microarray (TMA) builder 20010.02, Histopathology, Pécs, Hungary) for the immunohistochemical analysis. Further, three representative regions from peri- and intratumoral areas were selected on the same H&E sections (4 µm each), and marked for SHG methodology (Supplementary Information 1).

### Immunohistochemical staining and evaluation

TMA sections were deparaffinized, rehydrated and submitted to antigen retrieval. The primary antibodies used, dilutions and detection methods are briefly described in Supplementary Table 6. Seven cases of medullary carcinoma kindly provided by Camille Franchet were analyzed in whole sections. The evaluation of immunohistochemical results was centrally performed by two experienced breast pathologists (CAA and FCS).

Staining for estrogen-receptor alpha (ER), progesterone receptor (PR) and androgen receptor (AR) were evaluated according to the previously described guidelines^62^. For p63, keratin 5 and P-cadherin, samples were scored as positive when ≥10% cells were immunoreactive^63^. Membrane expression for HER-2 and EGFR was evaluated as previously described^64^. Ki-67 was evaluated by counting 1000 neoplastic cells in areas with highest positivity; cases with <20% positive cells were considered to have low proliferation, and the others, high proliferation^65^. Tumors were classified based on St Gallen Internation Expert Consensus^65^ (Supplementary Information 1).

### Second-harmonic generation (SHG) imaging

SHG microscopy was performed on a Zeiss LSM 780-NLO inverted confocal system (Carl Zeiss AG, Göttingen, Germany) available at the National Institute of Sciences and Technology on Photonics Applied to Cell Biology (INFABiC, UNICAMP) (Supplementary Information 1, Fig. 4 and Supplementary Fig. 7). Three peri- and three intratumoral areas selected for collagen assessment on H&E stained sections were examined in this condition, and digital images were collected for further evaluation. Peritumoral regions were defined as the fibrous tissue at the tumor borders, in the transition with non-neoplastic tissue. Intratumoral regions corresponded to fibrous bands within groups of neoplastic cells. In cases with multiple tumor sections, the most representative one was chosen.

**Figure 4:**
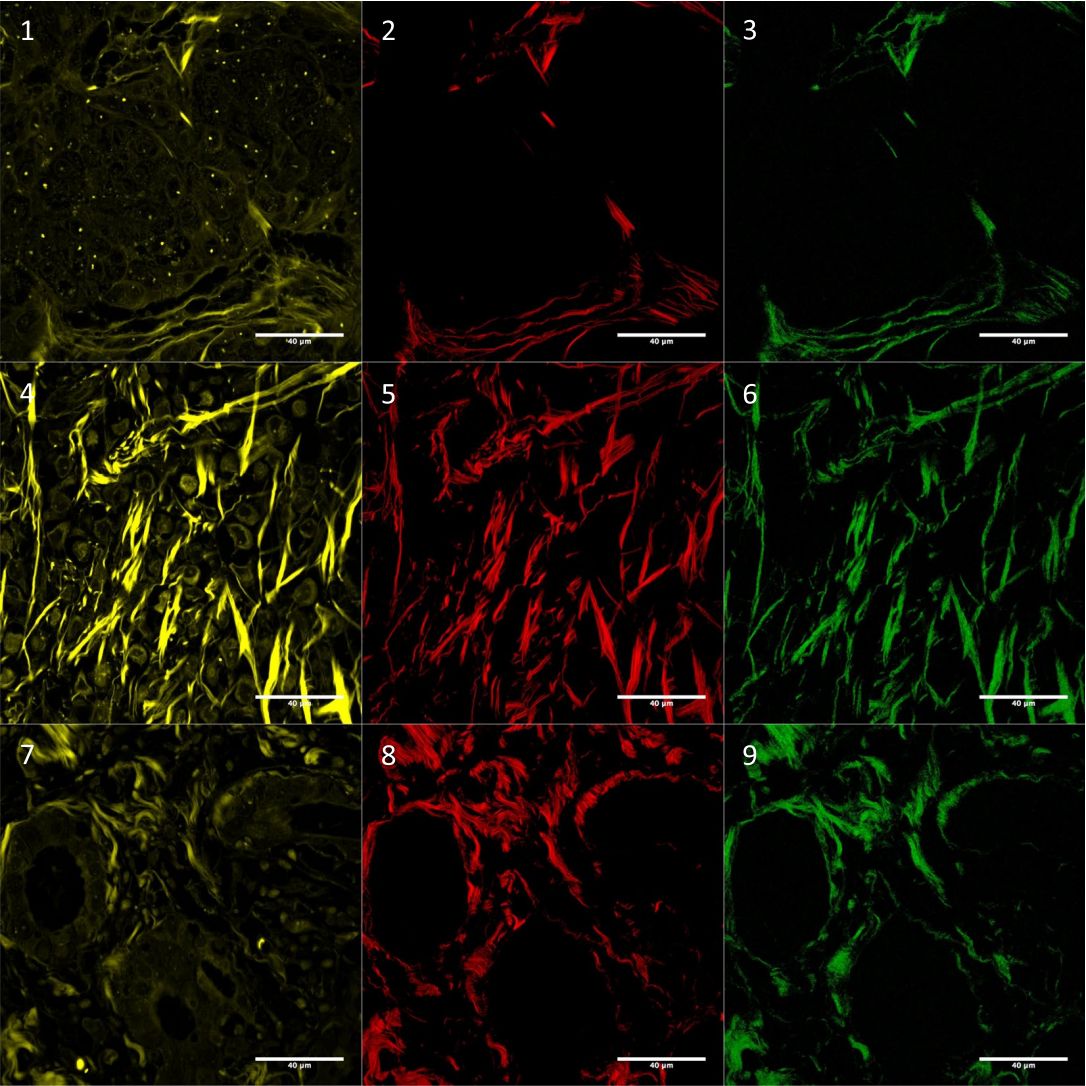
Autofluorescence (01, 04 and 07), fSHG collagen (02, 05 and 08) and bSHG collagen (03, 06 and 09). Images correspond to: invasive ductal carcinoma no special type (01, 02 and 03), classic invasive lobular (04, 05 and 06) and tubular (07, 08 and 09). Autofluorescence was included in this Figure to show tissue architecture. The images were log-transformed. For more examples see Supplementary Fig. 1. [bSHG, fSHG: backward and forward propagation second harmonic generation, respectively].

### Evaluation of collagen fibers

SHG images were stratified regarding collagen parameters using image pattern analysis methods. Collagen parameter refers to the pixels characterization in the SHG image, being evaluated through three features: quantity, uniformity and organization. Quantitative analysis of collagen parameters was performed in SHG images using ImageJ (http://imagej.nih.gov/ij/) and OrientationJ plug-in^66^. For this purpose, 16 representative areas to cover entire image (256 × 256 pixels) were performed in each image. The final value of each parameter represents the average of the values obtained in the three images; the value of each image denotes the average of the 16 representative areas of each image. (Supplementary Information 1). IBC-NST, being the most common subtype of breast cancer, was used as a comparison parameter for the other cases. Therefore, special cases may present collagen parameters equal, higher or lower than IBC-NST. Furthermore, a previous report demonstrated that the area of the cellular component does not represent a confounding variable in the collagen parameters evaluation^67^, so hypo- or hypercellular tumors were evaluated in the same way.

### Statistical analyses

Collagen quantity and uniformity were log-transformed to base *e* for statistical purposes; statistical analyses were performed using R (https://cran.r-project.org/). Shapiro-Wilk test was performed to analyze the data distribution of collagen parameters. Comparisons were performed using t-tests or analysis of variance (ANOVA). Pearson’s Correlation coefficients were calculated to analyze the correlation between collagen parameters. A multivariate recursive partitioning model was fit to determine groups of collagen between breast cancer subtypes. Hierarchical clustering analysis was performed, using Euclidean distance and Ward’s clustering algorithm^49,51,68^ and minimal spanning tree (Supplementary Information 1). *p* values were corrected using Benjamini and Hochberg adjustment to minimize α errors, and adjusted *p* values less than 0.05 were considered significant.

## Supplementary information

Supplementary info
