# Supplementary material for: Exploring Collagen Parameters in Pure Special Types of Invasive Breast Cancer
Source: Sci Rep. 2019 May 22;9:7715. doi: 10.1038/s41598-019-44156-9 (PMC6531485; doi:10.1038/s41598-019-44156-9)
Supplement: Supplementary file 1 — Supplementary info [file 41598_2019_44156_MOESM1_ESM.docx]

***Title***

**EXPLORING COLLAGEN PARAMETERS IN PURE SPECIAL TYPES OF INVASIVE BREAST CANCER**

***Running title***

Collagen Parameters and Breast Cancer

***Authors***

Rodrigo A Natal^a^; Geisilene R Paiva^b^; Vitor B Pelegati^c^; Ludwing Marenco^c^, César A Alvarenga^d^; Renato F Vargas^b^; Sophie F Derchain^e^; Luis O Sarian^e^; Camille Franchet^f^; Carlos L Cesar^c,g^; Fernando C Schmitt^h,i^; Britta Weigelt^j^; José Vassallo^a†^

^a^ Laboratory of Investigative and Molecular Pathology, CIPED – Faculty of Medical Sciences – State University of Campinas. Rua Tessália Vieira de Camargo, 126, Zip code: 13083-970 – Campinas, São Paulo, Brazil.

^b^ Laboratory of Specialized Pathology, LAPE – Faculty of Medical Sciences – State University of Campinas. Rua Tessália Vieira de Camargo, 126, Zip code: 13083-970 – Campinas, São Paulo, Brazil.

^c^ Department of Quantum Electronics –Institute of Physics “Gleb Wataghin” – State University of Campinas. Rua Sérgio Buarque de Holanda, 777, Zip code: 13083-859 – Campinas, São Paulo, Brazil.

^d^ Instituto de Patologia de Campinas (Private Laboratory). Av. Andrade Neves, 1801, Zip Code: 13070-000 – Campinas, São Paulo, Brazil.

^e^ Department of Obstetrics and Gynecology – Faculty of Medical Sciences – State University of Campinas. Rua Tessália Vieira de Camargo, 126, Zip code: 13083-970 – Campinas, São Paulo, Brazil.

^f^ Department of Pathology –University Cancer Institute. Avenue Irene Joliot Curie, 1, Zip code: 31059 – Toulousse, France.

^g^ Department of Physics, Federal University of Ceará (UFC), Campus do Pici - Bloco 922 - Zip code: 60455-760 – Fortaleza – Ceará, Brazil.

^h^ Institute of Molecular Pathology and Immunology of Porto University (IPATIMUP) – Porto University. Rua Dr. Roberto Frias, s/n, Zip code: 4200-465 – Porto, Portugal.

^i^ National Santé Laboratory, Department of Medicine – L-3555, Dudelange, Luxembourg.

^j^ Department of Pathology – Memorial Sloan Kettering Cancer Center. York Avenue 1275, Zip code: 10065 – New York, USA.

^†^ Correspondence to: José Vassallo MD, PhD or Rodrigo de Andrade Natal. Laboratory of Investigative and Molecular Pathology, CIPED – Faculty of Medical Sciences – State University of Campinas. Rua Tessália Vieira de Camargo, 126, Zip code: 13083-970 – Campinas, São Paulo, Brazil. E-mail: vassallomeister@gmail.com or rodrigo.natal.med@gmail.com. Phone: +55 19 3521-8987.

**Supplementary Table 1**: Correlations between collagen parameters

| Collagen parameters | Peritumoral bSHG collagen quantity | Peritumoral bSHG collagen uniformity |
| --- | --- | --- |
| Peritumoral bSHG collagen uniformity | r= 0.979; p< 0.001 | - |
| Peritumoral bSHG collagen organization | r= 0.238; p= 0.008 | r= 0.342; p< 0.001 |

| Collagen parameters | Peritumoral fSHG collagen quantity | Peritumoral fSHG collagen uniformity |
| --- | --- | --- |
| Peritumoral fSHG collagen uniformity | r= 0.957; p< 0.001 | - |
| Peritumoral fSHG collagen organization | r= 0.108; p= 0.241 | r= 0.091; p= 0.323 |

| Collagen parameters | Intratumoral bSHG collagen quantity | Intratumoral bSHG collagen uniformity | |
| --- | --- | --- | --- |
| Intratumoral bSHG collagen uniformity | r= 0.941; p< 0.001 | - |  |
| Intratumoral bSHG collagen organization | r= 0.819; p< 0.001 | r= 0. 902; p< 0.001 |  |

| Collagen parameters | Intratumoral fSHG collagen quantity | Intratumoral fSHG collagen uniformity | |
| --- | --- | --- | --- |
| Intratumoral fSHG collagen uniformity | r= 0.943; p< 0.001 | - |  |
| Intratumoral fSHG collagen organization | r= 0.888; p< 0.001 | r= 0.881; p< 0.001 |  |

A
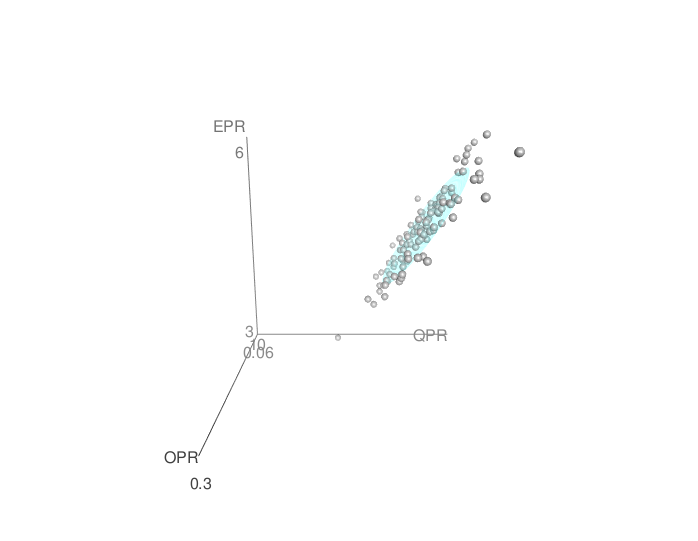
B
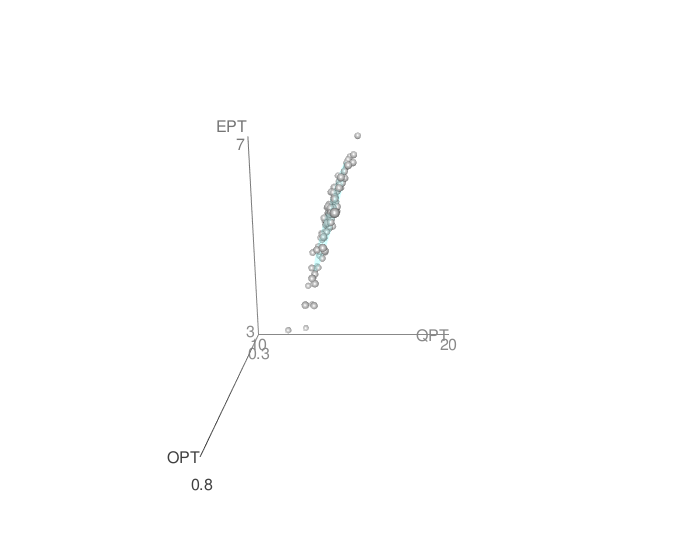


C
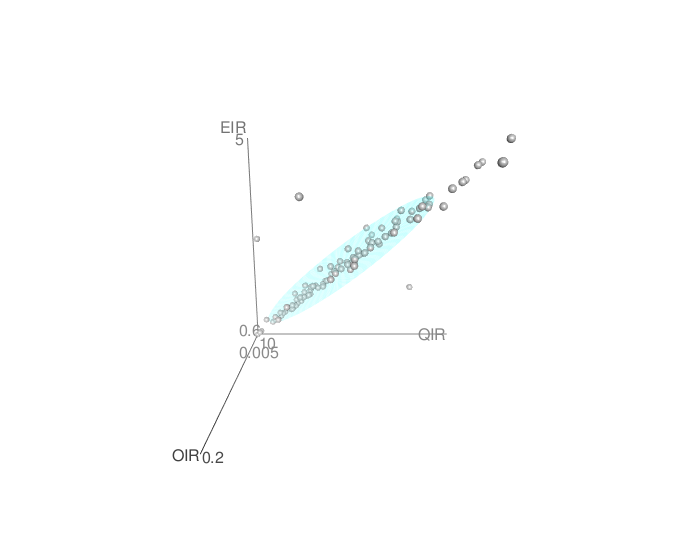
D
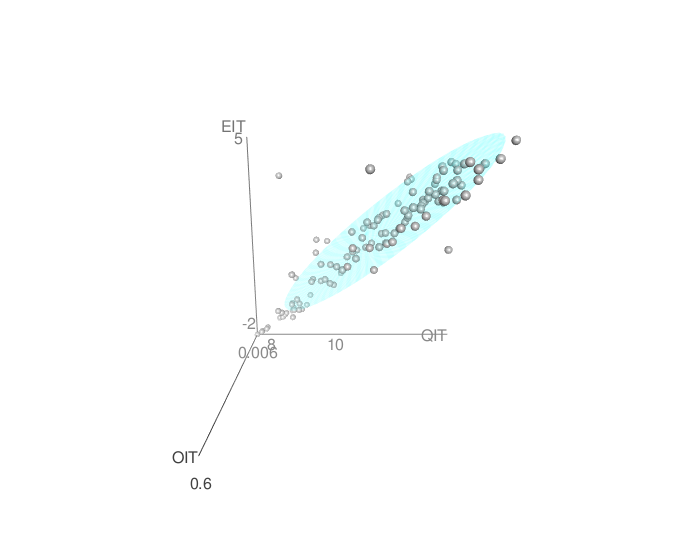


**Supplementary Figure 1 -** 3d-scatter plots demonstrate poor correlation between collagen parameters in: (A) Peritumoral bSHG collagen parameters (axis labels refer to peritumoral bSHG collagen quantity (QPR), uniformity (EPR) and organization (OPR)); and (B) Peritumoral fSHG collagen parameters (axis labels refer to peritumoral fSHG collagen quantity (QPT), uniformity (EPT) and organization (OPT)). Higher poor correlation between collagen parameter in: (C) Intratumoral bSHG collagen parameters (axis labels refer to intratumoral bSHG collagen quantity (QIR), uniformity (EIR) and organization (OIR)); and (D) Intratumoral fSHG collagen parameters (axis labels refer to intratumoral fSHG collagen quantity (QIT), uniformity (EIT) and organization (OIT)).

**Supplementary Table 2**: Comparisons between peri- and intratumoral collagen parameters.

|  | **BSHG collagen fibers** | | | | | | | | | **FSHG collagen fibers** | | | | | | | | |
| --- | --- | --- | --- | --- | --- | --- | --- | --- | --- | --- | --- | --- | --- | --- | --- | --- | --- | --- |
|  | **Quantity** | | | **Uniformity** | | | **Organization** | | | **Quantity** | | | **Uniformity** | | | **Organization** | | |
| **Histological Types** | **Peritumoral** | **Intratumoral** | ***p*** | **Peritumoral** | **Intratumoral** | ***p*** | **Peritumoral** | **Intratumoral** | ***p*** | **Peritumoral** | **Intratumoral** | ***p*** | **Peritumoral** | **Intratumoral** | ***p*** | **Peritumoral** | **Intratumoral** | ***p*** |
| IC-NST | 13.61 (0.42) | 11.68 (0.73) | <0.001 | 5.08 (0.50) | 2.86 (0.85) | <0.001 | 0.16 (0.04) | 0.08 (0.04) | <0.001 | 14.03 (0.48) | 11.55 (1.04) | <0.001 | 5.84 (0.70) | 2.86 (1.32) | <0.001 | 0.44 (0.07) | 0.36 (0.12) | 0.011 |
| Classic invasive lobular | 13.84 (0.46) | 12.21 (0.79) | 0.009 | 5.40 (0.56) | 3.60 (1.02) | 0.017 | **0.20 (0.04)** | **0.14 (0.06)** | **0.112** | 14.19 (0.52) | 12.00 (0.74) | 0.002 | 6.07 (0.69) | 3.44 (0.74) | 0.001 | **0.48 (0.08)** | **0.46 (0.07)** | **0.202** |
| Tubular | 13.85 (0.34) | 12.31 (0.66) | <0.001 | 5.35 (0.39) | 3.50 (0.90) | <0.001 | 0.17 (0.05) | 0.11 (0.04) | 0.002 | 14.05 (0.35) | 12.13 (0.82) | <0.001 | 5.82 (0.47) | 3.58 (1.18) | <0.001 | **0.48 (0.08)** | **0.41 (0.10)** | **0.121** |
| Mucinous | 13.58 (0.42) | 10.80 (0.77) | <0.001 | 5.11 (0.52) | 1.93 (1.01) | <0.001 | 0.15 (0.04) | 0.04 (0.04) | <0.001 | 13.85 (0.43) | 9.83 (1.68) | <0.001 | 5.67 (0.58) | 0.99 (2.37) | <0.001 | 0.43 (0.08) | 0.18 (0.16) | <0.001 |
| Papillary | 13.39 (0.31) | 10.64 (0.17) | <0.001 | 4.81 (0.38) | 1.45 (0.25) | <0.001 | 0.16 (0.05) | 0.01 (0.02) | <0.001 | 13.75 (0.56) | 9.21 (0.95) | <0.001 | 5.37 (0.92) | -0.42 (1.54) | <0.001 | 0.44 (0.08) | 0.09 (0.12) | <0.001 |
| Micropapillary | 13.74 (0.52) | 11.64 (0.50) | <0.001 | 5.24 (0.56) | 3.21 (0.61) | 0.003 | **0.17 (0.06)** | **0.10 (0.04)** | **0.070** | 14.12 (0.52) | 11.40 (0.76) | <0.001 | 5.94 (0.58) | 3.03 (1.04) | <0.001 | **0.49 (0.17)** | **0.44 (0.11)** | **0.607** |
| Medullary | 13.33 (0.51) | 10.73 (0.41) | <0.001 | 4.82 (0.58) | 1.67 (0.53) | <0.001 | 0.16 (0.04) | 0.04 (0.03) | <0.001 | 13.79 (0.65) | 9.83 (1.14) | <0.001 | 5.67 (0.81) | 0.83 (1.73) | <0.001 | 0.44 (0.05) | 0.22 (0.14) | <0.001 |
| Metaplastic | 13.20 (0.47) | 11.26 (0.43) | <0.001 | 4.64 (0.52) | 2.28 (0.52) | <0.001 | 0.15 (0.03) | 0.05 (0.04) | 0.003 | 13.75 (0.43) | 11.21 (0.62) | <0.001 | 5.59 (0.47) | 2.43 (1.02) | <0.001 | **0.42 (0.05)** | **0.33 (0.13)** | **0.071** |
| Apocrine | 13.47 (0.35) | 11.30 (0.74) | <0.001 | 4.89 (0.42) | 2.44 (0.94) | <0.001 | 0.15 (0.03) | 0.07 (0.04) | <0.001 | 13.87 (0.30) | 11.01 (1.23) | <0.001 | 5.63 (0.49) | 2.35 (1.60) | <0.001 | 0.45 (0.09) | 0.34 (0.13) | 0.007 |
| **Total** | 13.5 (0.45) | 11.3  (0.84) | <0.001 | 5.03 (0.52) | 2.46 (1.05) | <0.001 | 0.16 (0.04) | 0.07 (0.05) | <0.001 | 13.9 (0.47) | 10.8 (1.47) | <0.001 | 5.72 (0.64) | 1.98 (1.95) | <0.001 | 0.45 (0.08) | 0.30 (0.17) | <0.001 |

^†^ Data in: Mean (Standard deviation).

AB

CD

EF

**Supplementary Figure 2**: Boxplots demonstrating distribution of peritumoral collagen parameters by histological subtypes: (A) bSHG collagen quantity; (B) fSHG collagen quantity; (C) bSHG collagen uniformity; (D) fSHG collagen uniformity; (E) bSHG collagen oranization; and (F) fSHG collagen organization. All histological subtypes: invasive ductal carcinoma of no special type (1. IC-NST), classic invasive lobular (2. cILC), tubular (3. TUB), mucinous (4. MUC), papillary (5. PAP), micropapillary (6. mPAP), medullary (7. MED), metaplastic (8. METAP), and invasive apocrine (9. APO) presented the same values for peritumoral collagen parameters.

**Supplementary Table 3**: Comparisons between peri- and intratumoral collagen parameters and histological subtypes.

| **Collagen parameters** | **IC-NST** | **Classic invasive lobular** | **Tubular** | **Mucinous** | **Papillary** | **Micropapillary** | **Medullary** | **Metaplasic** |
| --- | --- | --- | --- | --- | --- | --- | --- | --- |
| **Peritumoral bSHG collagen quantity** | |  |  |  |  |  |  |  |
| Classic invasive lobular | 0.341 |  |  |  |  |  |  |  |
| Tubular | 0.233 | 0.954 |  |  |  |  |  |  |
| Mucinous | 0.886 | 0.310 | 0.178 |  |  |  |  |  |
| Papillary | 0.341 | 0.135 | 0.080 | 0.388 |  |  |  |  |
| Micropapillary | 0.599 | 0.729 | 0.687 | 0.548 | 0.244 |  |  |  |
| Medullary | 0.157 | 0.080 | **0.028** | 0.178 | 0.780 | 0.157 |  |  |
| Metaplastic | 0.080 | **0.040** | **0.021** | 0.092 | 0.479 | 0.080 | 0.581 |  |
| Apocrine | 0.479 | 0.157 | 0.080 | 0.548 | 0.720 | 0.338 | 0.479 | 0.244 |
| **Peritumoral fSHG collagen quantity** | |  |  |  |  |  |  |  |
| Classic invasive lobular | 0.760 |  |  |  |  |  |  |  |
| Tubular | 0.920 | 0.810 |  |  |  |  |  |  |
| Mucinous | 0.430 | 0.370 | 0.430 |  |  |  |  |  |
| Papillary | 0.370 | 0.370 | 0.370 | 0.830 |  |  |  |  |
| Micropapillary | 0.860 | 0.910 | 0.910 | 0.430 | 0.370 |  |  |  |
| Medullary | 0.370 | 0.370 | 0.370 | 0.900 | 0.910 | 0.370 |  |  |
| Metaplastic | 0.370 | 0.370 | 0.370 | 0.830 | 0.980 | 0.370 | 0.910 |  |
| Apocrine | 0.540 | 0.370 | 0.540 | 0.910 | 0.810 | 0.520 | 0.830 | 0.810 |
| **Peritumoral bSHG collagen uniformity** | |  |  |  |  |  |  |  |
| Classic invasive lobular | 0.277 |  |  |  |  |  |  |  |
| Tubular | 0.277 | 0.886 |  |  |  |  |  |  |
| Mucinous | 0.901 | 0.281 | 0.277 |  |  |  |  |  |
| Papillary | 0.281 | 0.085 | 0.079 | 0.277 |  |  |  |  |
| Micropapillary | 0.650 | 0.682 | 0.735 | 0.682 | 0.275 |  |  |  |
| Medullary | 0.277 | 0.079 | 0.066 | 0.237 | 0.963 | 0.237 |  |  |
| Metaplastic | 0.085 | 0.050 | **0.042** | 0.085 | 0.611 | 0.085 | 0.528 |  |
| Apocrine | 0.346 | 0.085 | 0.079 | 0.281 | 0.757 | 0.277 | 0.757 | 0.323 |
| **Peritumoral fSHG collagen uniformity** | |  |  |  |  |  |  |  |
| Classic invasive lobular | 0.690 |  |  |  |  |  |  |  |
| Tubular | 0.950 | 0.690 |  |  |  |  |  |  |
| Mucinous | 0.690 | 0.690 | 0.720 |  |  |  |  |  |
| Papillary | 0.690 | 0.690 | 0.690 | 0.690 |  |  |  |  |
| Micropapillary | 0.880 | 0.880 | 0.880 | 0.690 | 0.690 |  |  |  |
| Medullary | 0.690 | 0.690 | 0.760 | 0.990 | 0.690 | 0.690 |  |  |
| Metaplastic | 0.690 | 0.690 | 0.690 | 0.880 | 0.720 | 0.690 | 0.880 |  |
| Apocrine | 0.690 | 0.690 | 0.690 | 0.930 | 0.690 | 0.690 | 0.930 | 0.940 |
| **Peritumoral bSHG collagen organization** | |  |  |  |  |  |  |  |
| Classic invasive lobular | 0.550 |  |  |  |  |  |  |  |
| Tubular | 0.910 | 0.690 |  |  |  |  |  |  |
| Mucinous | 0.690 | 0.240 | 0.690 |  |  |  |  |  |
| Papillary | 0.970 | 0.550 | 0.910 | 0.850 |  |  |  |  |
| Micropapillary | 0.910 | 0.690 | 0.970 | 0.690 | 0.910 |  |  |  |
| Medullary | 0.970 | 0.550 | 0.910 | 0.720 | 0.970 | 0.910 |  |  |
| Metaplastic | 0.720 | 0.310 | 0.690 | 0.970 | 0.860 | 0.690 | 0.760 |  |
| Apocrine | 0.690 | 0.240 | 0.690 | 0.970 | 0.850 | 0.690 | 0.720 | 0.970 |
| **Peritumoral fSHG collagen organization** | |  |  |  |  |  |  |  |
| Classic invasive lobular | 0.710 |  |  |  |  |  |  |  |
| Tubular | 0.710 | 0.930 |  |  |  |  |  |  |
| Mucinous | 0.930 | 0.710 | 0.710 |  |  |  |  |  |
| Papillary | 0.950 | 0.710 | 0.710 | 0.930 |  |  |  |  |
| Micropapillary | 0.710 | 0.930 | 0.930 | 0.710 | 0.710 |  |  |  |
| Medullary | 0.930 | 0.710 | 0.710 | 0.910 | 0.930 | 0.710 |  |  |
| Metaplastic | 0.860 | 0.710 | 0.710 | 0.910 | 0.910 | 0.710 | 0.800 |  |
| Apocrine | 0.910 | 0.750 | 0.750 | 0.800 | 0.910 | 0.710 | 0.830 | 0.710 |
| **Intratumoral bSHG collagen quantity** | |  |  |  |  |  |  |  |
| Classic invasive lobular | 0.097 |  |  |  |  |  |  |  |
| Tubular | **0.015** | 0.789 |  |  |  |  |  |  |
| Mucinous | **< 0.001** | **< 0.001** | **< 0.001** |  |  |  |  |  |
| Papillary | **< 0.001** | **< 0.001** | **< 0.001** | 0.606 |  |  |  |  |
| Micropapillary | 0.906 | 0.155 | 0.068 | **0.013** | **0.011** |  |  |  |
| Medullary | **< 0.001** | **< 0.001** | **< 0.001** | 0.789 | 0.789 | **0.011** |  |  |
| Metaplastic | 0.144 | **0.011** | **0.001** | 0.106 | 0.074 | 0.318 | 0.086 |  |
| Apocrine | 0.107 | **0.007** | **< 0.001** | **0.034** | **0.029** | 0.318 | **0.029** | 0.906 |
| **Intratumoral fSHG collagen quantity** | |  |  |  |  |  |  |  |
| Classic invasive lobular | 0.473 |  |  |  |  |  |  |  |
| Tubular | 0.258 | 0.839 |  |  |  |  |  |  |
| Mucinous | **< 0.001** | **< 0.001** | **< 0.001** |  |  |  |  |  |
| Papillary | **< 0.001** | **< 0.001** | **< 0.001** | 0.260 |  |  |  |  |
| Micropapillary | 0.827 | 0.461 | 0.280 | **0.009** | **0.002** |  |  |  |
| Medullary | **< 0.001** | **< 0.001** | **< 0.001** | 0.987 | 0.280 | **0.012** |  |  |
| Metaplastic | 0.559 | 0.263 | 0.125 | **0.008** | **0.001** | 0.827 | **0.012** |  |
| Apocrine | 0.258 | 0.112 | **0.021** | **0.005** | **< 0.001** | 0.562 | **0.011** | 0.764 |
| **Intratumoral bSHG collagen uniformity** | |  |  |  |  |  |  |  |
| Classic invasive lobular | 0.068 |  |  |  |  |  |  |  |
| Tubular | 0.068 | 0.786 |  |  |  |  |  |  |
| Mucinous | **0.001** | **< 0.001** | **< 0.001** |  |  |  |  |  |
| Papillary | **< 0.001** | **< 0.001** | **< 0.001** | 0.185 |  |  |  |  |
| Micropapillary | 0.427 | 0.429 | 0.520 | **0.002** | **< 0.001** |  |  |  |
| Medullary | **< 0.001** | **< 0.001** | **< 0.001** | 0.417 | 0.561 | **< 0.001** |  |  |
| Metaplastic | 0.115 | **0.004** | **0.003** | 0.355 | 0.062 | 0.062 | 0.117 |  |
| Apocrine | 0.160 | **0.005** | **0.002** | 0.086 | **0.009** | 0.082 | **0.020** | 0.659 |
| **Intratumoral fSHG collagen uniformity** | |  |  |  |  |  |  |  |
| Classic invasive lobular | 0.555 |  |  |  |  |  |  |  |
| Tubular | 0.609 | 0.841 |  |  |  |  |  |  |
| Mucinous | **0.001** | **0.002** | **< 0.001** |  |  |  |  |  |
| Papillary | **< 0.001** | **< 0.001** | **< 0.001** | 0.053 |  |  |  |  |
| Micropapillary | 0.841 | 0.761 | 0.841 | **0.018** | **< 0.001** |  |  |  |
| Medullary | **0.001** | **0.002** | **< 0.001** | 0.841 | 0.124 | **0.016** |  |  |
| Metaplastic | 0.618 | 0.351 | 0.371 | 0.052 | **0.001** | 0.609 | **0.044** |  |
| Apocrine | 0.489 | 0.228 | 0.228 | 0.228 | **< 0.001** | 0.524 | **0.023** | 0.898 |
| **Intratumoral bSHG collagen organization** | |  |  |  |  |  |  |  |
| Classic invasive lobular | **0.003** |  |  |  |  |  |  |  |
| Tubular | 0.191 | 0.083 |  |  |  |  |  |  |
| Mucinous | **0.001** | **< 0.001** | **< 0.001** |  |  |  |  |  |
| Papillary | **< 0.001** | **< 0.001** | **< 0.001** | 0.129 |  |  |  |  |
| Micropapillary | 0.362 | 0.129 | 0.931 | **0.002** | **< 0.001** |  |  |  |
| Medullary | **0.002** | **< 0.001** | **< 0.001** | 0.808 | 0.210 | **0.002** |  |  |
| Metaplastic | 0.073 | **< 0.001** | **0.006** | 0.515 | 0.76 | **0.029** | 0.438 |  |
| Apocrine | 0.516 | **0.001** | 0.073 | **0.017** | **0.001** | 0.187 | **0.017** | 0.203 |
| **Intratumoral fSHG collagen organization** | |  |  |  |  |  |  |  |
| Classic invasive lobular | 0.137 |  |  |  |  |  |  |  |
| Tubular | 0.361 | 0.507 |  |  |  |  |  |  |
| Mucinous | **< 0.001** | **< 0.001** | **< 0.001** |  |  |  |  |  |
| Papillary | **< 0.001** | **< 0.001** | **< 0.001** | 0.095 |  |  |  |  |
| Micropapillary | 0.241 | 0.821 | 0.666 | **< 0.001** | **< 0.001** |  |  |  |
| Medullary | **0.003** | **< 0.001** | **< 0.001** | 0.546 | **0.040** | **0.001** |  |  |
| Metaplastic | 0.560 | 0.077 | 0.186 | **0.014** | **< 0.001** | 0.142 | 0.077 |  |
| Apocrine | 0.641 | 0.077 | 0.186 | **< 0.001** | **< 0.001** | 0.142 | **0.018** | 0.821 |

**Supplementary Table 4**: Peri- and intratumoral collagen parameters compared with histological grade

| **Histological grade** | **Grade I** | **Grade II** |
| --- | --- | --- |
| **Peritumoral** |  |  |
| **BSHG collagen quatity** |  |  |
| Grade II | 0.311 |  |
| Grade III | **0.008** | 0.140 |
| **FSHG collagen quatity** |  |  |
| Grade II | 0.400 |  |
| Grade III | 0.310 | 0.740 |
| **BSHG collagen uniformity** |  |  |
| Grade II | 0.196 |  |
| Grade III | **0.009** | 0.197 |
| **FSHG collagen uniformity** |  |  |
| Grade II | 0.540 |  |
| Grade III | 0.540 | 0.740 |
| **BSHG collagen organization** |  |  |
| Grade II | 0.460 |  |
| Grade III | 0.860 | 0.460 |
| **FSHG collagen organization** |  |  |
| Grade II | 0.990 |  |
| Grade III | 0.330 | 0.330 |
| **Intratumoral** |  |  |
| **BSHG collagen quatity** |  |  |
| Grade II | 0.245 |  |
| Grade III | **0.003** | 0.097 |
| **FSHG collagen quatity** |  |  |
| Grade II | 0.330 |  |
| Grade III | 0.220 | 0.720 |
| **BSHG collagen uniformity** |  |  |
| Grade II | 0.196 |  |
| Grade III | **0.005** | 0.196 |
| **FSHG collagen uniformity** |  |  |
| Grade II | 0.270 |  |
| Grade III | 0.270 | 0.640 |
| **BSHG collagen organization** |  |  |
| Grade II | 0.410 |  |
| Grade III | 0.150 | 0.410 |
| **FSHG collagen organization** |  |  |
| Grade II | 0.830 |  |
| Grade III | 0.830 | 0.830 |

**Supplementary Table 5**: Peri- and intratumoral collagen parameters compared with immunohistochemical-based subtypes

a. Collagen parameters in invasive breast carcinoma

| **Immunohistochemical-based subtype** | **HR+ HER2-** | **HR+ HER2+** | **HR- HER2+** | **HR- HER2-** |
| --- | --- | --- | --- | --- |
| **Peritumoral** |  |  |  |  |
| FSHG collagen quantity^†^ | 13.92 (0.50) | 13.96 (0.29) | 13.95 (0.06) | 13.78 (0.52) |
| BSHG collagen quantity^†^ | 13.61 (0.39) | 13.56 (0.22) | 13.11 (0.31) | 13.35 (0.51) |
| FSHG collagen uniformity^†^ | 5.70 (0.69) | 5.84 (0.34) | 5.81 (0.28) | 5.63 (0.63) |
| BSHG collagen uniformity^†^ | 5.09 (0.46) | 5.06 (0.25) | 4.47 (0.42) | 4.82 (0.57) |
| FSHG collagen organization^†^ | 0.46 (0.08) | 0.42 (0.07) | 0.43 (0.07) | 0.43 (0.06) |
| BSHG collagen organization^†^ | 0.16 (0.05) | 0.17 (0.03) | 0.14 (0.03) | 0.16 (0.04) |
| **Intratumoral** |  |  |  |  |
| FSHG collagen quantity^†^ | 10.89 (1.56) | 11.73 (0.81) | 10.07 (1.53) | 10.47 (1.20) |
| BSHG collagen quantity^†^ | 11.42 (0.89) | 11.80 (0.58) | 10.54 (0.51) | 10.97 (0.59) |
| FSHG collagen uniformity^†^ | 2.06 (2.00) | 3.72 (0.86) | 1.08 (1.83) | 1.66 (1.68) |
| BSHG collagen uniformity^†^ | 2.58 (1.08) | 3.56 (0.42) | 1.44 (00.61) | 2.00 (0.74) |
| FSHG collagen organization^†^ | 0.30 (0.17) | 0.48 (0.07) | 0.24 (0.13) | 0.26 (0.15) |
| BSHG collagen organization^†^ | 0.07 (0.05) | 0.13 (0.03) | 0.03 (0.02) | 0.05 (0.04) |

^†^ Data in: Mean (Standard deviation).

b. Comparisons between collagen parameters and immunohistochemical-based subtypes

| **Collagen parameters** | **HR+ HER2-** | **HR+ HER2+** | **HR- HER2+** |
| --- | --- | --- | --- |
| **Peritumoral bSHG collagen quantity** |  |  |  |
| HR+ HER2+ | 0.835 |  |  |
| HR- HER2+ | 0.079 | 0.273 |  |
| HR- HER2- | 0.072 | 0.422 | 0.422 |
| **Peritumoral fSHG collagen quantity** |  |  |  |
| HR+ HER2+ | 0.999 |  |  |
| HR- HER2+ | 0.999 | 0.999 |  |
| HR- HER2- | 0.999 | 0.999 | 0.999 |
| **Peritumoral bSHG collagen uniformity** |  |  |  |
| HR+ HER2+ | 0.897 |  |  |
| HR- HER2+ | 0.065 | 0.185 |  |
| HR- HER2- | 0.065 | 0.437 | 0.281 |
| **Peritumoral fSHG collagen uniformity** |  |  |  |
| HR+ HER2+ | 0.900 |  |  |
| HR- HER2+ | 0.900 | 0.900 |  |
| HR- HER2- | 0.900 | 0.950 | 0.900 |
| **Peritumoral bSHG collagen organization** |  |  |  |
| HR+ HER2+ | 0.640 |  |  |
| HR- HER2+ | 0.640 | 0.640 |  |
| HR- HER2- | 0.640 | 0.640 | 0.640 |
| **Peritumoral fSHG collagen organization** |  |  |  |
| HR+ HER2+ | 0.970 |  |  |
| HR- HER2+ | 0.970 | 0.970 |  |
| HR- HER2- | 0.420 | 0.970 | 0.970 |
| **Intratumoral bSHG collagen quantity** |  |  |  |
| HR+ HER2+ | 0.367 |  |  |
| HR- HER2+ | 0.070 | 0.070 |  |
| HR- HER2- | 0.070 | 0.087 | 0.367 |
| **Intratumoral fSHG collagen quantity** |  |  |  |
| HR+ HER2+ | 0.330 |  |  |
| HR- HER2+ | 0.330 | 0.320 |  |
| HR- HER2- | 0.610 | 0.330 | 0.320 |
| **Intratumoral bSHG collagen uniformity** |  |  |  |
| HR+ HER2+ | 0.276 |  |  |
| HR- HER2+ | **0.038** | **0.009** |  |
| HR- HER2- | 0.276 | **0.031** | **0.009** |
| **Intratumoral fSHG collagen uniformity** |  |  |  |
| HR+ HER2+ | 0.570 |  |  |
| HR- HER2+ | 0.460 | 0.460 |  |
| HR- HER2- | 0.570 | 0.460 | 0.150 |
| **Intratumoral bSHG collagen organization** |  |  |  |
| HR+ HER2+ | 0.513 |  |  |
| HR- HER2+ | 0.085 | 0.144 |  |
| HR- HER2- | 0.513 | 0.085 | **0.022** |
| **Intratumoral fSHG collagen organization** |  |  |  |
| HR+ HER2+ | 0.793 |  |  |
| HR- HER2+ | 0.538 | 0.585 |  |
| HR- HER2- | 0.081 | 0.081 | 0.081 |

**Supplementary Figure 3**: Graphic representation of a multivariate response recursive partitioning model to determine the cutoffs for intratumoral collagen quantity, uniformity and organization.


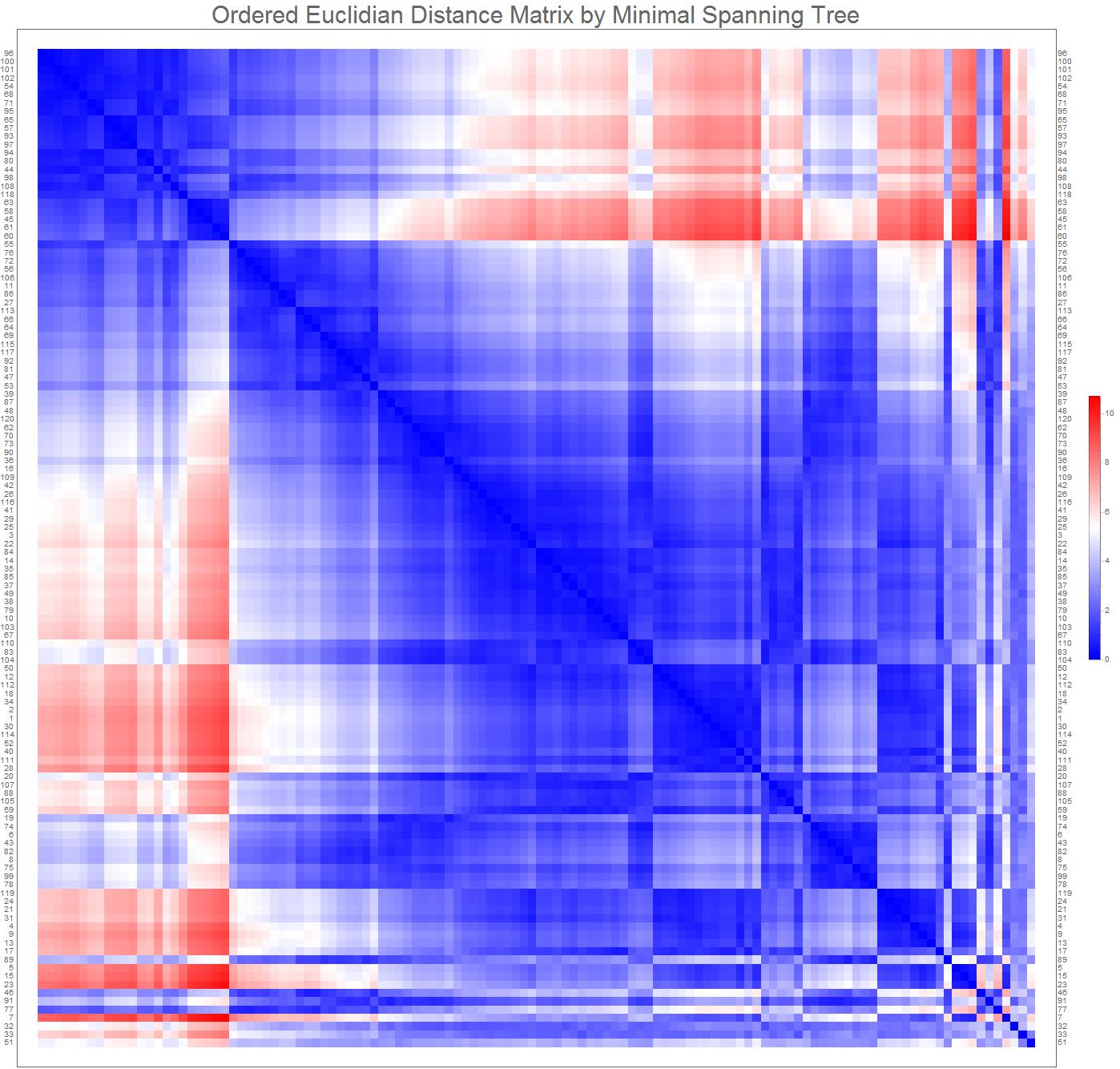


**Supplementary Figure 4**: Ordered Euclidean matrix by MST. We created a color scale on the matrix. Bluish colors represent high values of Euclidian distance and reddish colors are associated with lower values. The rearrangement of the matrix was made taking the values of weights of each pair of vertices of MST and ordering them in ascending values.


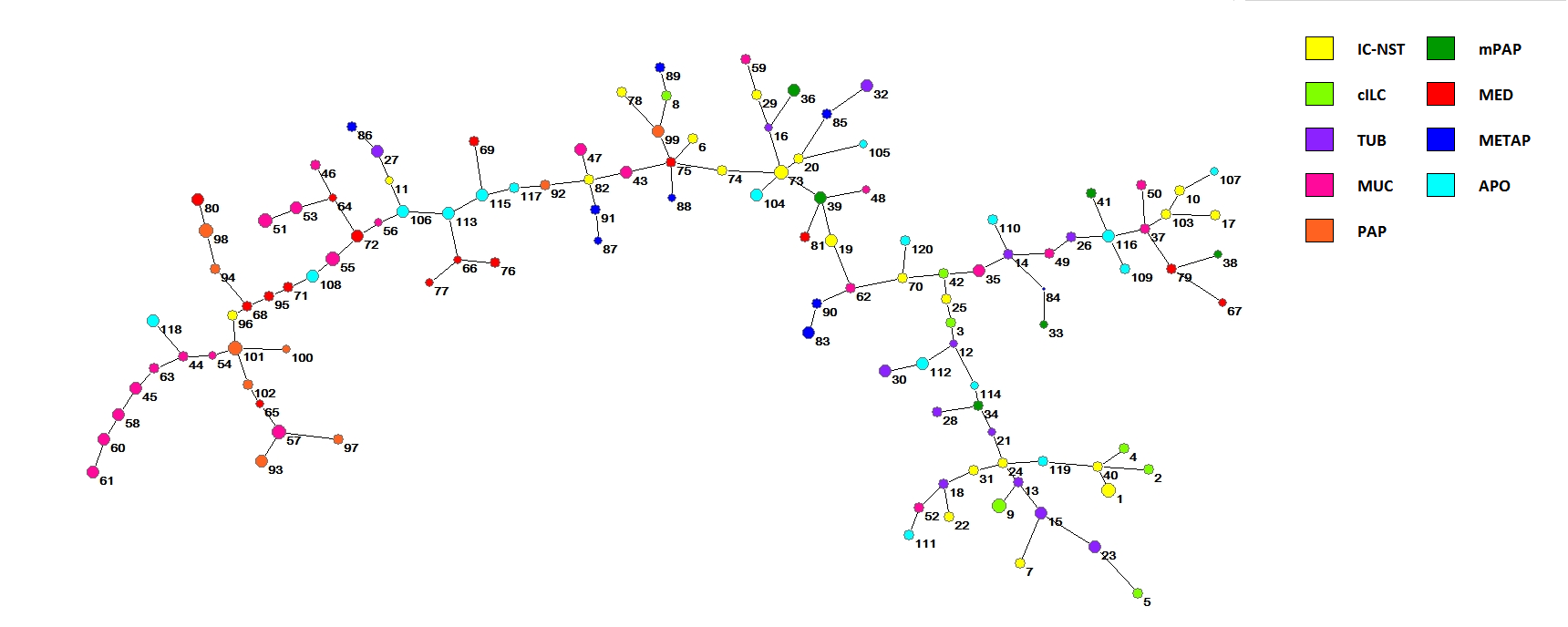


**Supplementary Figure 5**: Minimal Spanning Tree of the Euclidean matrix. Each different color represents one of the nine histological types of breast cancer; besides, the size of vertices corresponds to the ratio between the age of each patient and the maximum age of each group.

**Supplementary information 1:**

**Second-harmonic generation (SHG) imaging**

*Second-harmonic Generation Microscopy setup*

A 40x/1.3 oil immersion EC Plan-Neofluar objective (Carl Zeiss AG, Göttingen, Germany) was used. High numerical aperture (1.3) was necessary to provide the spatial resolution to observe the fibrils. The field of view of this objective was 212 x 212 μm. To avoid the edges of the picture, where the fluorescence is weaker, we used a digital zoom of 1.3 to choose an area of 163.5 x 163.5 μm, which was homogeneous. An excitation wavelength of 800 nm, with an approximately 100-fs width pulse at an 80-MHz repetition rate, was provided by a Mai-Tai^®^ Ti:Sapphire laser (Spectra-Physics, Irvine, CA, USA). Laser power in the objective lens incident on the sample was around 80 mW, with circular polarization. Acquisition time of each image was around 60 s. The resolution was approximately 0.3 µm.

SHG forward signal at 400nm was collected with a 0.55 NA – WD 26 mm condenser lens and detected by a photomultiplier tube (PMT). A short-pass SP690 (Omega Filters, Brattleboro, VT, USA) was used to filter the 800-nm excitation ^1^. It was followed by a filter cube composed of a LP490 dichroic mirror at 45 degrees and a SP405 filter at 0 degrees, to reflect only the SHG signal to the PMT. SHG backward signal, also at 400nm, was detected by the LSM780 internal PMT from 371-405nm.

The forward signal emitted by the sample, besides having SHG forward signal, also allowed captures the two-photons excited fluorescence signal, mainly generated by the eosin coloration. This signal was detected by a non-descanned (NDD). The split of these signals was possible due to the filter cube composed by a LP490 dichroic mirror at 45 degree. These images were not used for evaluation of the samples, but they identify the tumor area that we are evaluating.

We utilized the same procedure described by Burke and colleagues ^2^ to take into account day-to-day variations in optical alignments, comprising the acquisition of a normalization factor, provided by the SHG image of a standard sample (human aorta) at the beginning of each experimental session. To eliminate background, a blinded observer applied a common threshold to all images to distinguish collagen pixels from background pixels. Of 968 images, 950 were suitable for analysis.

**Immunohistochemical-based subtype**

Immunohistochemical-based subtype was based on hormone receptor (ER and PR) and HER2 expression, according to simplified St Gallen International Expert Consensus ^3^, thus:

| Clinical group | Acronym | Definition |
| --- | --- | --- |
| Hormone receptor positive and HER2 negative ^§^ | HR+ HER2- | ER and/or PR positive and HER2 negative |
| Luminal A like | LumA | High hormone receptor expression and low Ki-67 expression (< 20%) |
| Luminal B like | LumB | Low hormone receptor expression and high Ki-67 index (≥ 20%) |
| Hormone receptor positive and HER2 positive ^§^ | HR+ HER2+ | ER and/or PR positive and HER2 positive |
| Hormone receptor negative and HER2 positive ^§^ | HR- HER2+ | ER and PR negative and HER2 positive |
| Hormone receptor negative and HER2 negative ^§^ | HR- HER2- | ER and PR negative and HER2 negative |
| Basal-like | BL | EGFR positive, p63 positive, P-cadherin positive and/or keratin 5 positive |
| Non-basal-like | NBL | EGFR negative, p63 negative, P-cadherin negative and keratin 5 negative |

^§^ For immunohistochemical interpretation, Allred guidelines was followed ^4^, considering scores 6 to 8 as high hormone receptor expression ^5^. Apocrine invasive breast carcinoma corresponded to the grouping of pure apocrine and apocrine-like invasive carcinoma ^6^.

**Evaluation of collagen fibers**

*Image analysis*

Each image with 1024 x 1024 pixels was subdivided in 16 areas with 256 x 256 pixels to cover the entire image, like the following image.

For each image, the final collagen parameter value considered was the mean value of the 16 areas (Formula 1):

$\bar{x}=\frac{1}{n}\sum_{i=1}^{n} x_{i}$ 1

For each region of each sample, the mean value of the 3 final collagen parameter values was considered.

Since collagen fibril diameter in tissues (10 – 300 nm) is much smaller that the optical resolution, they are not resolved in the SHG images and it remains difficult to link the detected SHG signal to the collagen arrangement at the fibrillar scale ^7^. Backward SHG images exhibit punctate distribution attributable to small-diameter, segmental collagen. By contrast, images from the mature fibrils are identical in the forward and backward directions. Fibrillogenesis can be resolved in immature tissue by directly imaging backward-propagating SHG ^8^.

*Integrated Density* was performed in the ImageJ software to analyze the quantity of SHG signal and, thus, indirectly, the amount of collagen fibers. Using OrientationJ plug-in ^9^, evaluation of *Energy* (uniformity) and *Coherency* (organization) was performed. High uniformity values correspond to less isotropic and more clearly oriented collagen fibers; in other words, high values of uniformity indicate more orientated and nearer collagen fibers, producing bundles of collagen. Thus, superposition of collagen fibers in this case could limit the evaluation of other parameters. In addition, organization is bounded between 0 and 1; values tending to 1 indicate highly oriented collagen fibers structures. Fiber length size ranged from 5 to 10 µm.

A
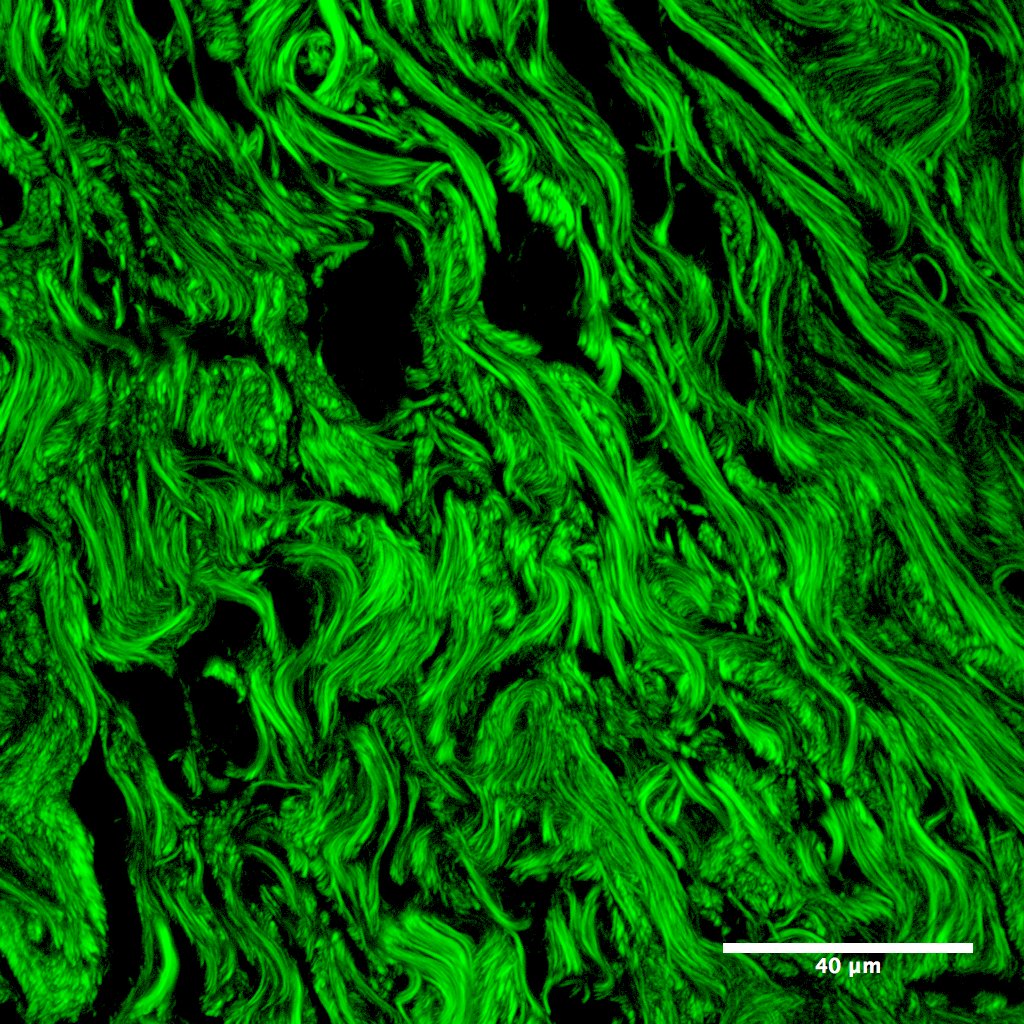
 B
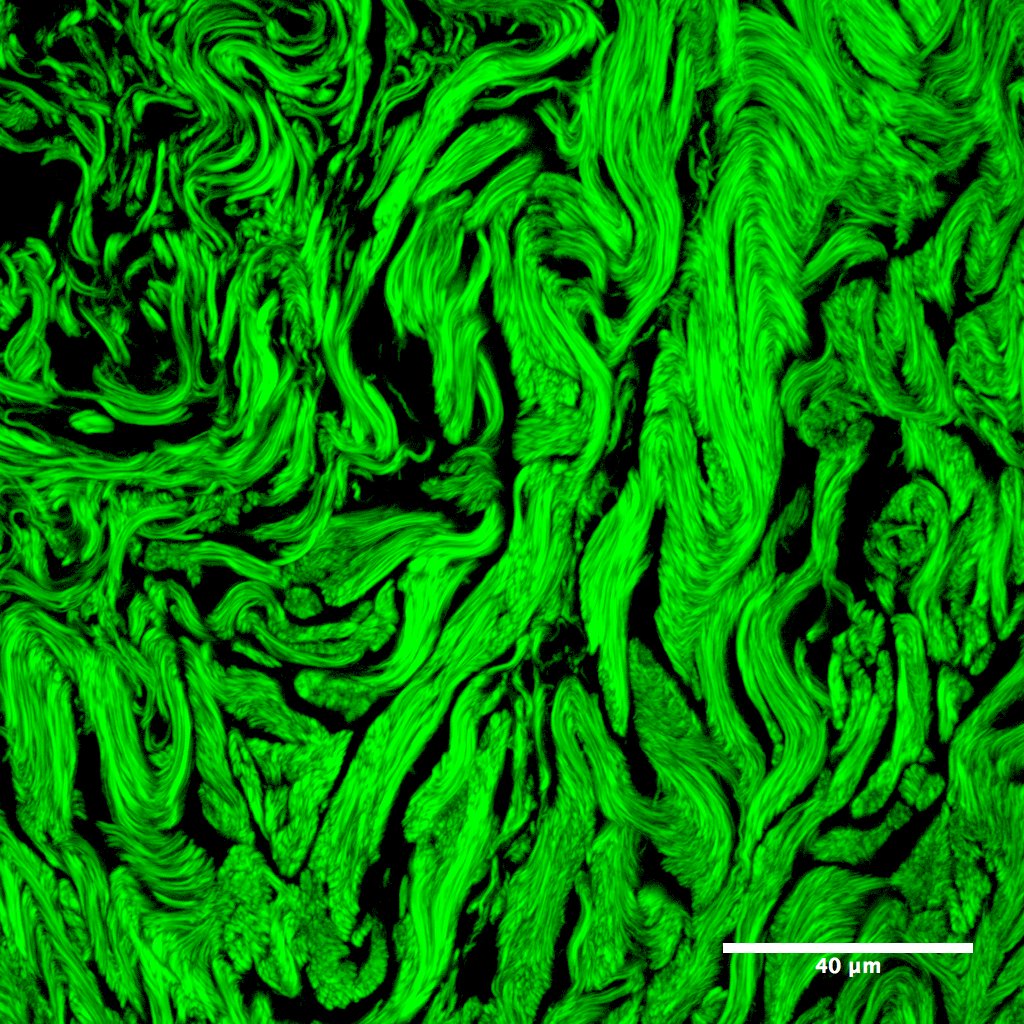
 C
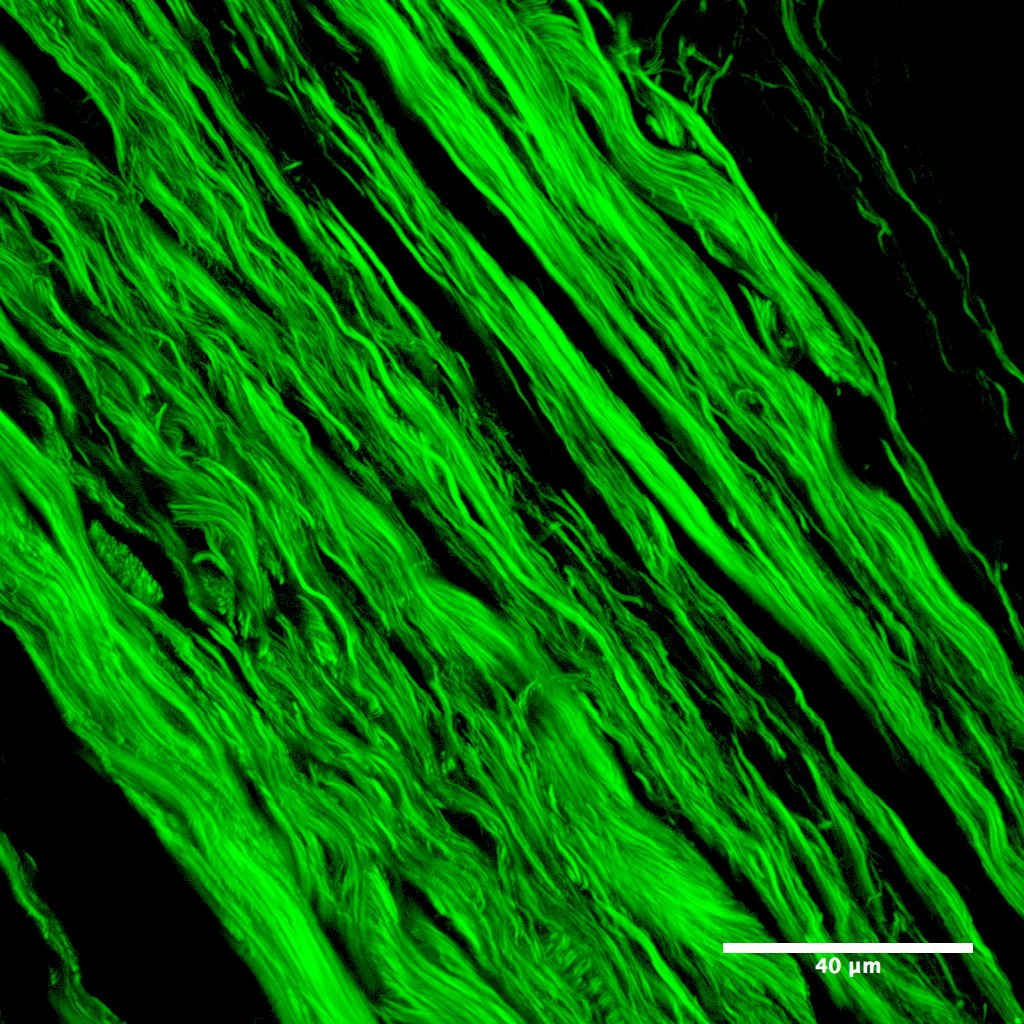


D
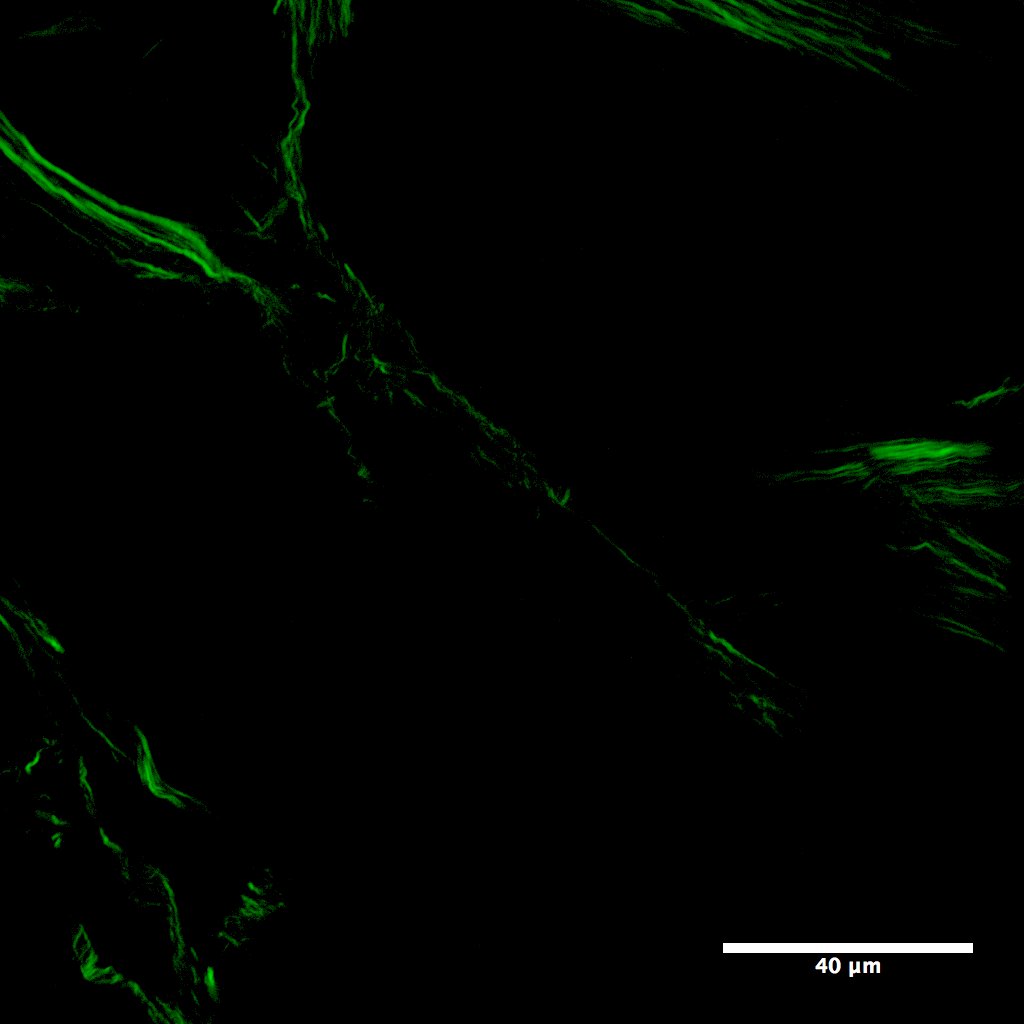
 E
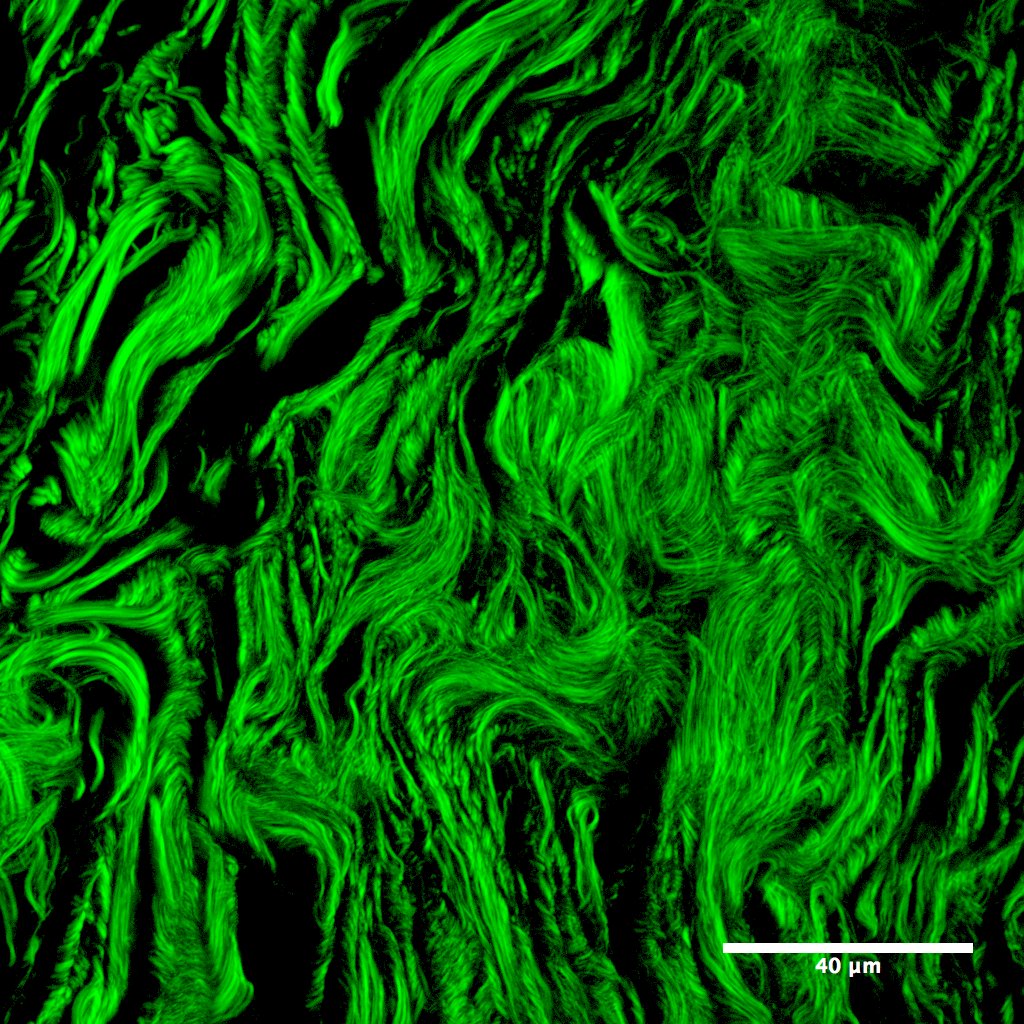
 F
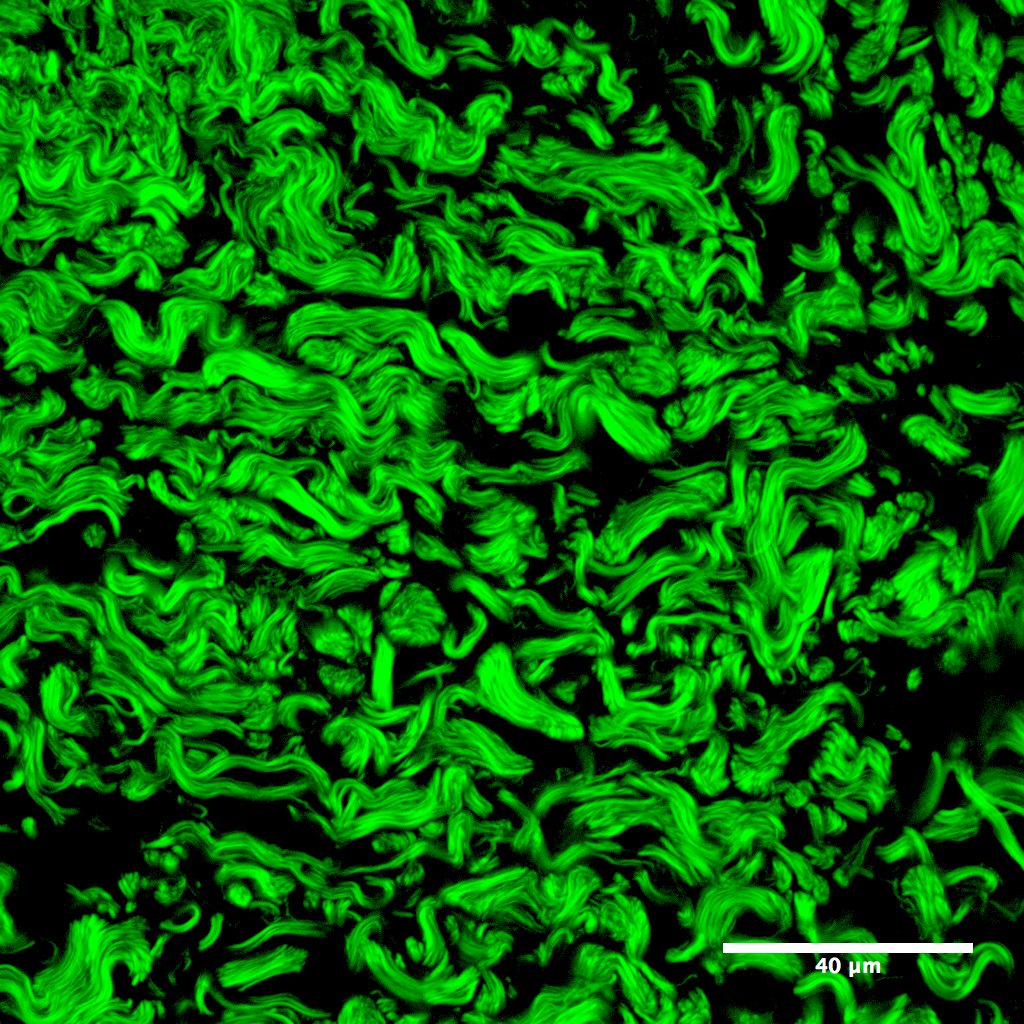


**Supplementary Figure 6**: Representative images of collagen parameters evaluated in this study. A and D: quantity; B and E: uniformity; C and F: organization. High values are found in images A, B and C; low values, in D, E and F. The images were log-transformed.

Concerning SHG, it has been demonstrated that this approach can detect primarily types I and II collagen (both form consisting of aligned fibers), and myosin within actomyosin complexes. Collagen types III and IV do not produce adequate SHG signals for imaging *in vivo* ^10,11^. However, recent studies have detected the signal emission by collagen type III *in vitro* ^12^. This might represent a bias when only one type of fiber is targeted. However, type III collagen is present mainly in blood vessels, uterus and bowel; type II collagen is known to deposit only in matrix-producing (cartilaginous) metaplastic carcinomas ^13^. Myosin and microtubules present enough organization to generate SHG signals, comparable to type I collagen fibers, only in muscle and neurons. Also, these elements can be observed mostly in the absence of collagen fibers ^14,15^. Thus, it is reasonable to assume that SHG signals detected in breast cancer tissue studied herein originate mostly from type I collagen.

For each sample, 6 areas were evaluated (3 intratumoral areas and 3 peritumoral areas), each area resulted in 2 images (based on SHG forward signal, for fSHG fibers, and SHG backward signal, for bSHG fibers). The final values were calculated as the mean of the three values for each type of signals: (1) peritumoral fSHG fibers, (2) intratumoral fSHG fibers, (3) peritumoral bSHG fibers, (4) intratumoral bSHG fibers.

**Statistical analyses**

*Minimal spanning tree*

A symmetry network was created using the Euclidean distance matrix with Pajek free software (http://mrvar.fdv.uni-lj.si/pajek/). The quantity of vertices is equivalent to whole group of patients studied. An edge between each pair of vertices is equal to element d_ij_ of the Euclidian distance matrix. An optimization process on the network was made by the extraction of its minimal spanning tree of the Euclidean network using a MVA on Excel (Microsoft Corporation, Albuquerque, NM, USA). The minimal spanning tree (MST) is a subgraph of the network that contains all vertices constrained to the sum of the weights of all edges between each pair of vertices must be minimum. The aim to compute the MST is to observe the grouping of patients taking into account intratumoral collagen parameters. The advantage of MST resides in enabling us to appreciate proximities among patients with different histological types of cancer according to intratumoral collagen parameters.

As MST contains all vertices of the Euclidian matrix, and these vertices are joined by the minimum weight possible, it was used to rearrange the Euclidean matrix by permutations on the rows and columns. Since the result of the MST is a list of initial and final vertices and their corresponding weight, the permutation on the matrix was made placing the vertices in ascending order according to the result of the MST. Thus, the first two columns and rows of the matrix correspond to the lowest weight of MST, the next two columns and rows correspond to the second lower value, and so on. In other words, all pairs of vertices were ordered in ascending order according to the MST weight. A color scale was created, where bluish colors represent lower values of Euclidian distance and reddish colors corresponding to high values. This rearrangement allowed us to identify groups with a considerable proximity, like it was obtained by MST.

**Supplementary Table 6**: Primary antibodies used in the present study

| Primary antibody | Clone | Dilution | Antigen retrieval | Scoring system |
| --- | --- | --- | --- | --- |
| Estrogen receptor | SP1 | 1:100 | Citrate, pH 6.0 | CAP ^16^ |
| Progesterone receptor | 1A6 | 1:40 | Citrate, pH 6.0 | CAP ^16^ |
| HER-2/neu | SP3 | 1:80 | Citrate, pH 6.0 | Score 0 and 1+ = Negative  Score 2+ = Equivocal  Score 3+ = Positive ^17^ |
| EGFR | 31G7 | 1:100 | 10 min, at pepsin | Score 0 and 1+ = Negative  Score 2+ and 3+ = Positive ^17^ |
| Keratin 5 | XM26 | 1:50 | EDTA, pH 8.6 | ≥ 10% ^18^ |
| P-cadherin | 56 | 1:50 | EDTA, pH 8.6 | ≥ 10% ^18^ |
| P63 protein | 4A4 | 1:150 | Citrate, pH 6.0 | ≥ 10% ^18^ |
| Androgen receptor | AR27 | 1:50 | EDTA, pH 8.6 | CAP ^16^ |
| Ki-67 | SP6 | 1:200 | Citrate, pH 6.0 | < 14% = Low  ≥ 14% = High ^19^ |

Suppliers: Clones SP1, SP3, XM26, 4A4 and SP6: LabVision/ Neomarkers, Fremont, California, USA; Clones 1A6 and AR27: Novocastra, Newcastle upon Tyne, UK; Clone 31G7, Invitrogen/ Zymed, S Francisco, California, USA; Clone 56, Becton-Dickson (BD) Transduction, S Jose, California, USA. Detection method streptavidin-biotin-complex, LabVision (for SP3, XM26 and 4A4), and horseradish peroxidase-polymer, Dako, Glostrup, Denmark (for all other markers).

01
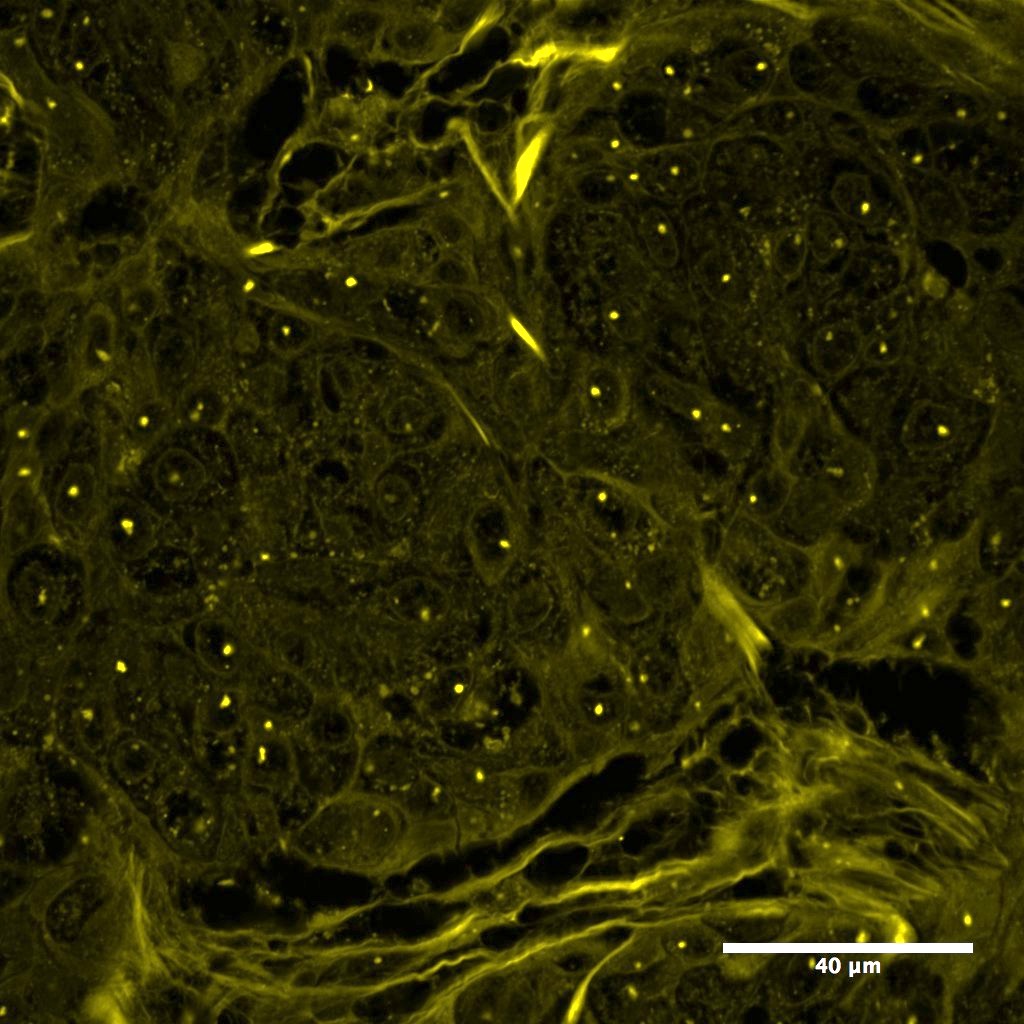
02
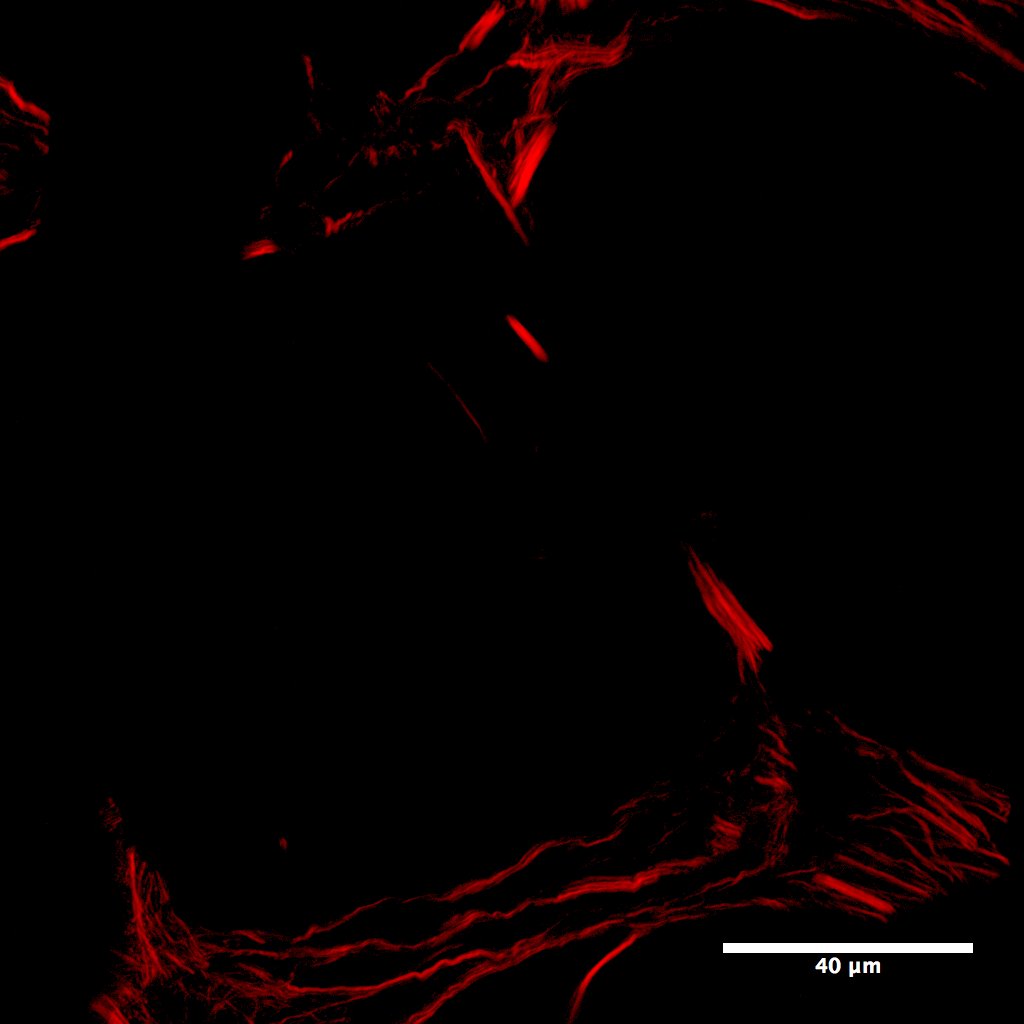
03
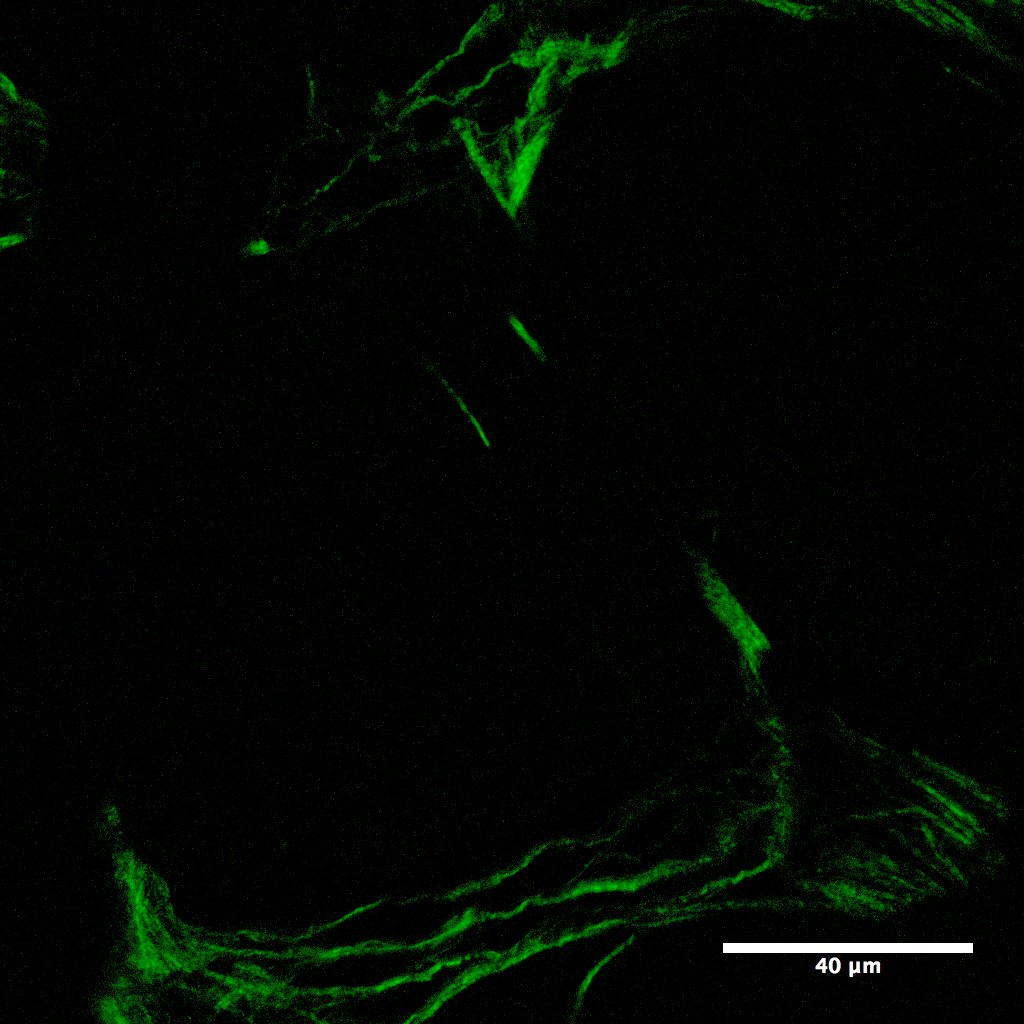
04
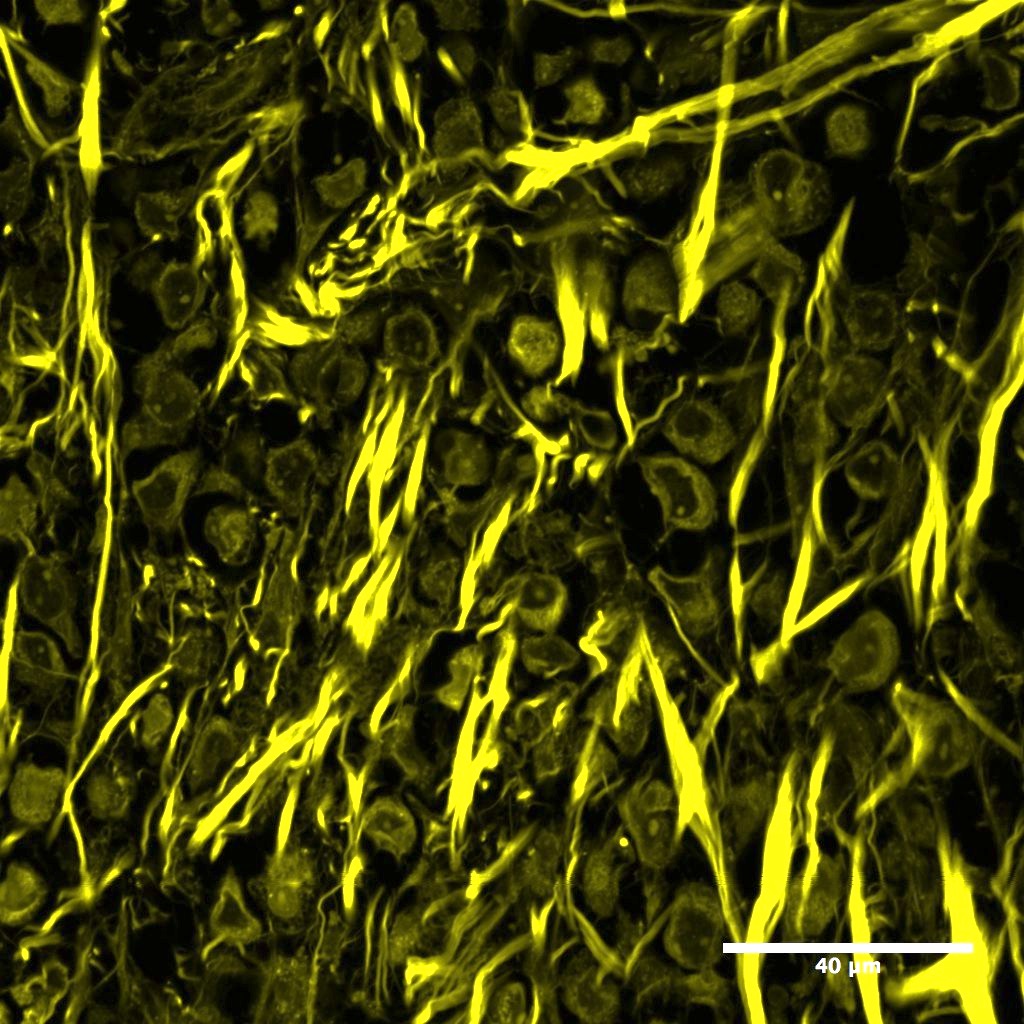
05
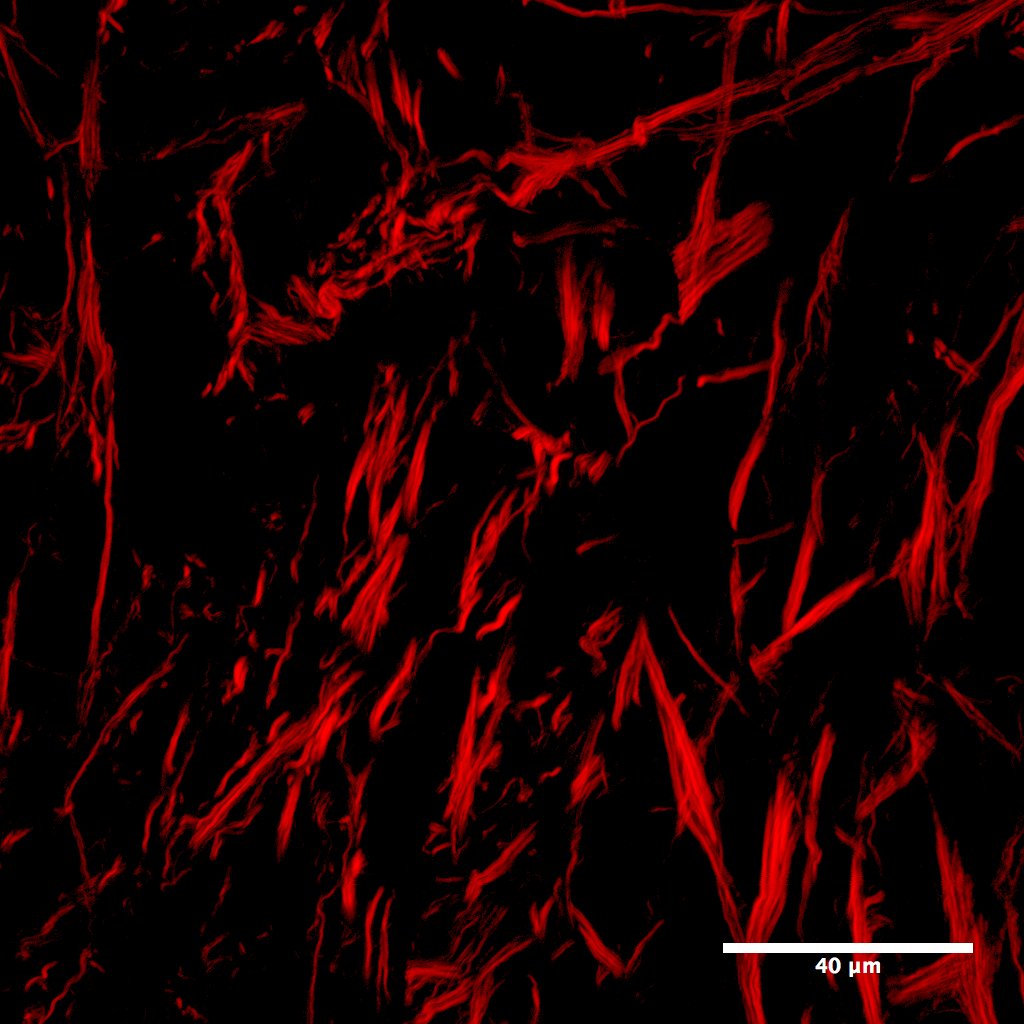
06
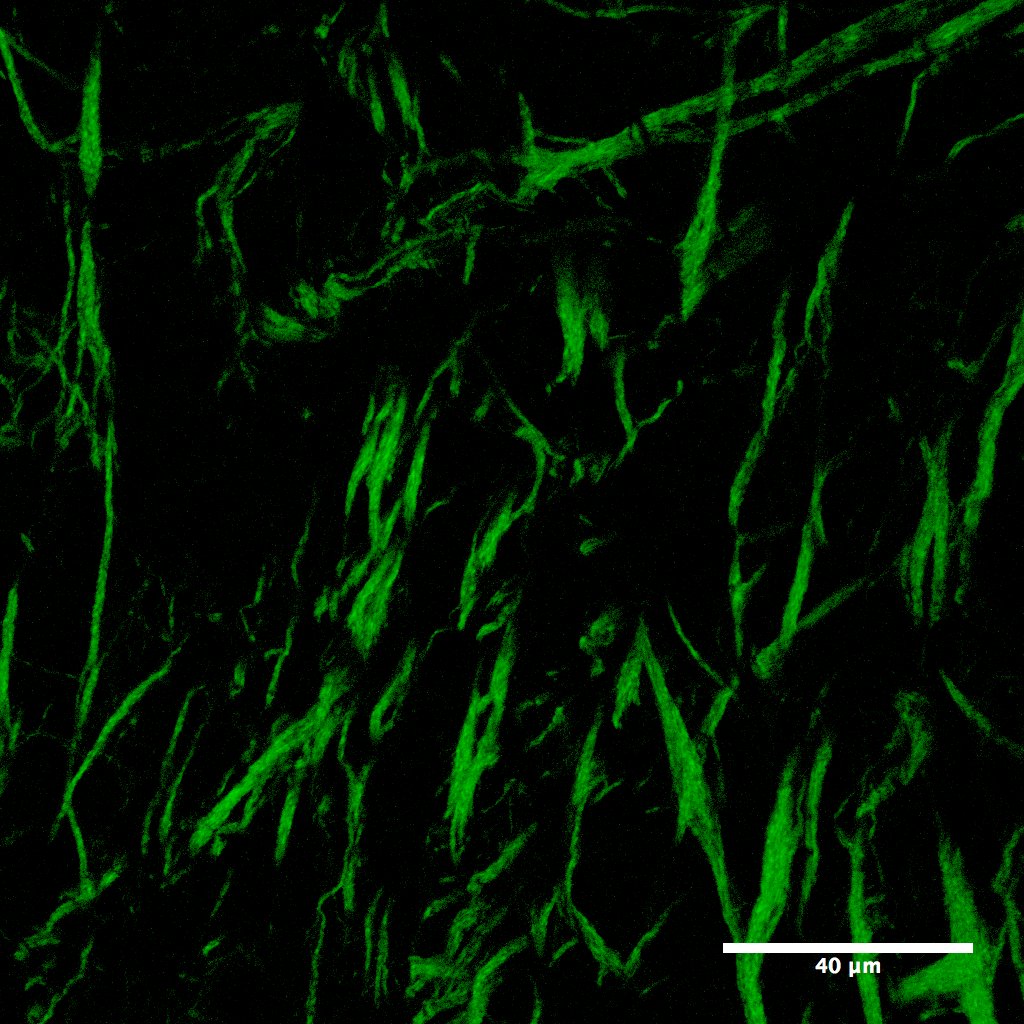


07
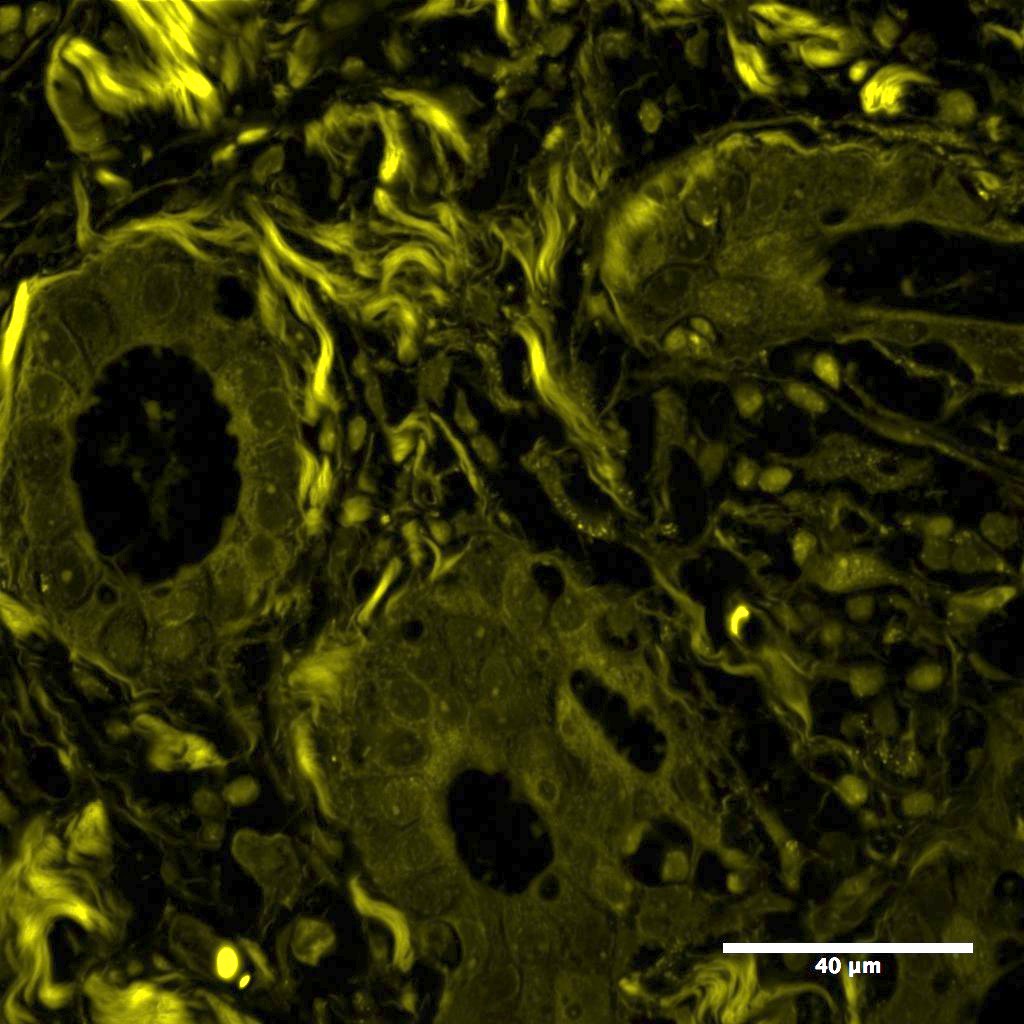
08
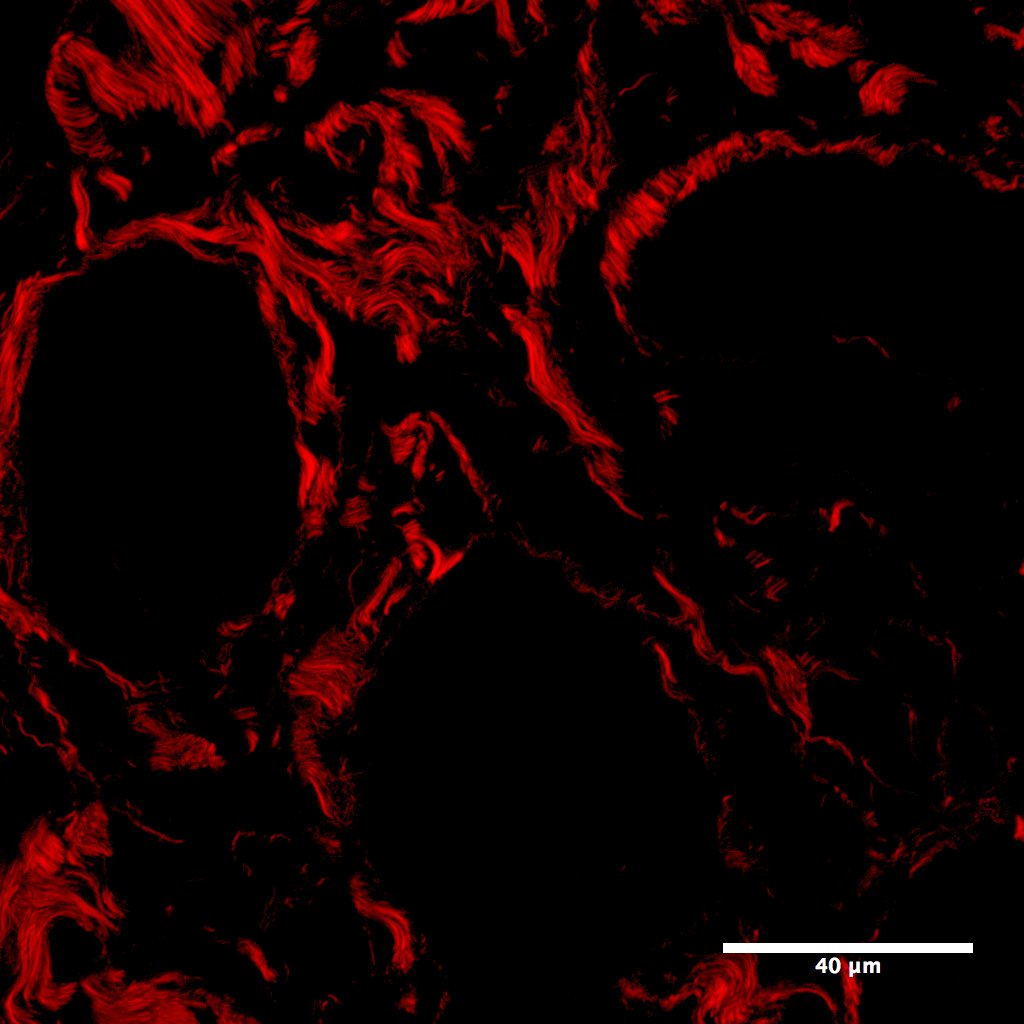
09
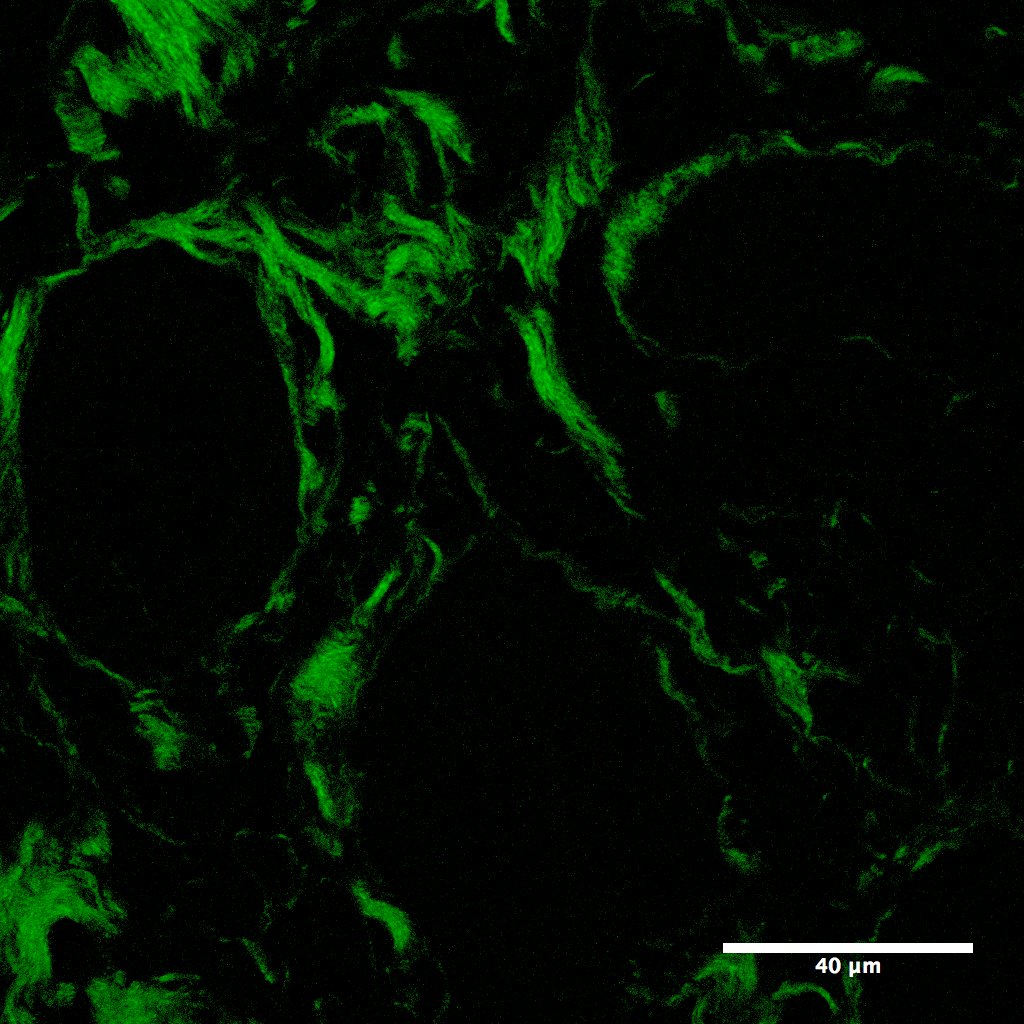
10
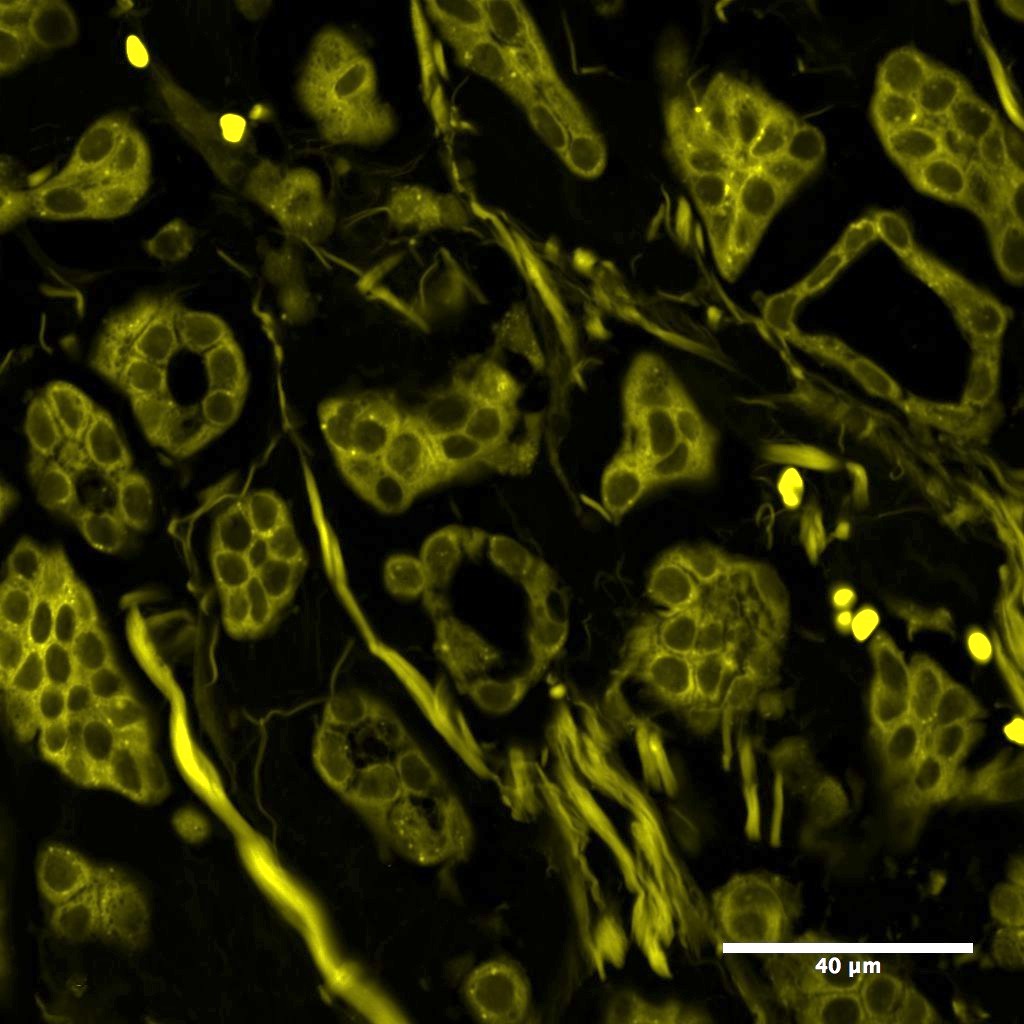
11
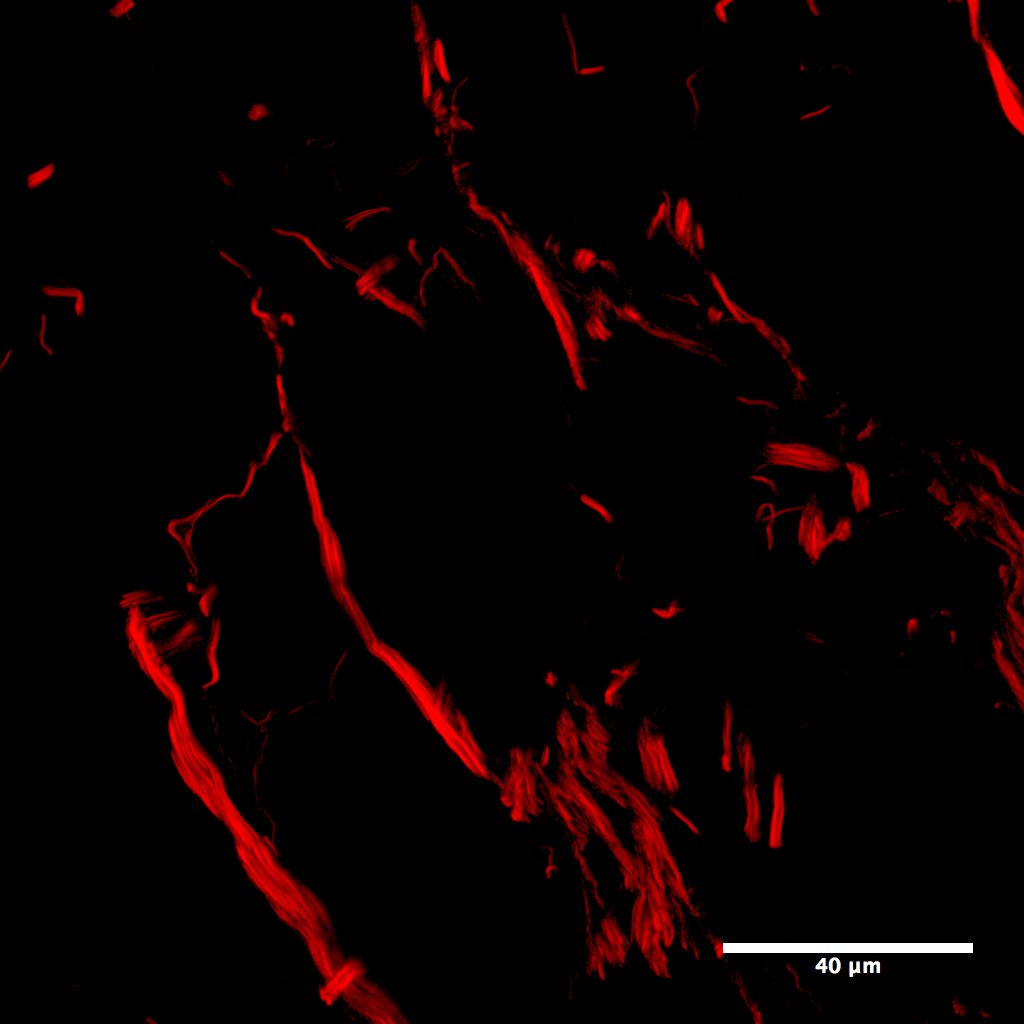
12
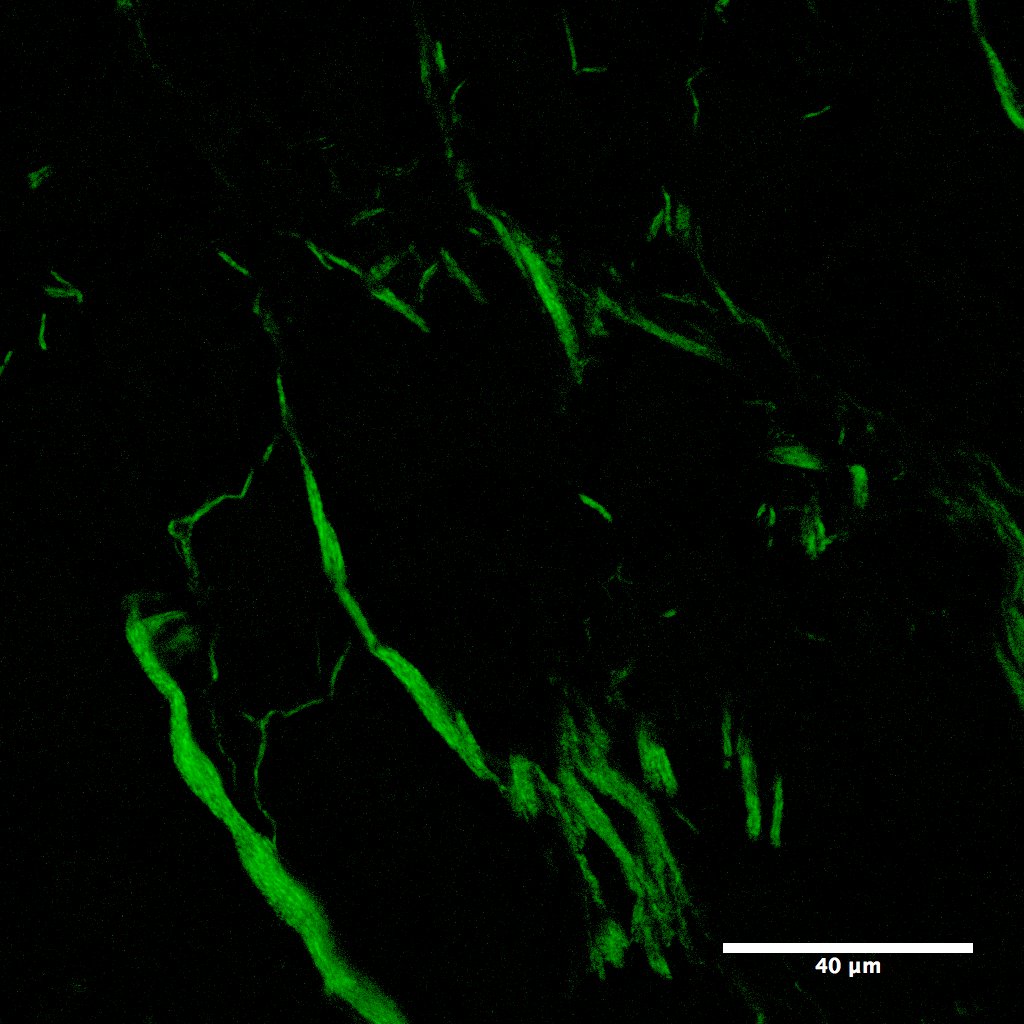


13
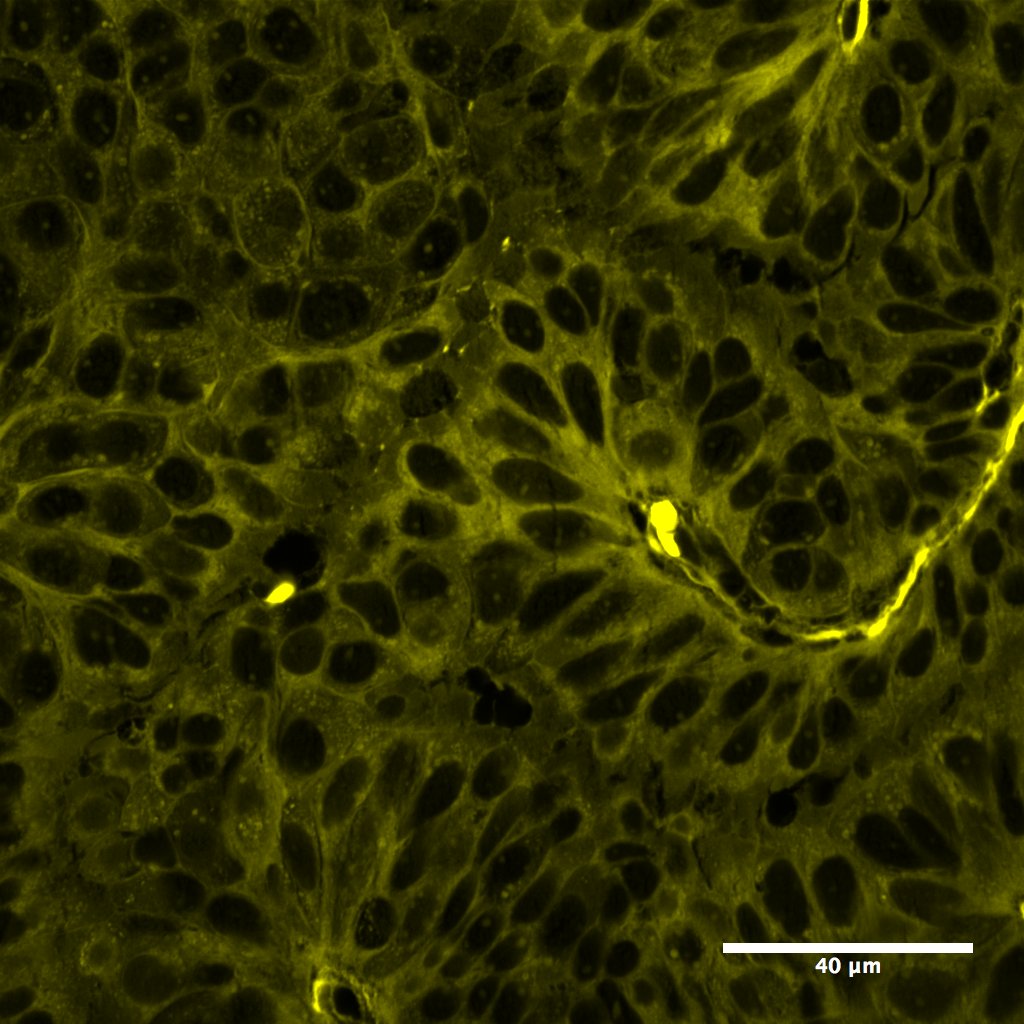
14
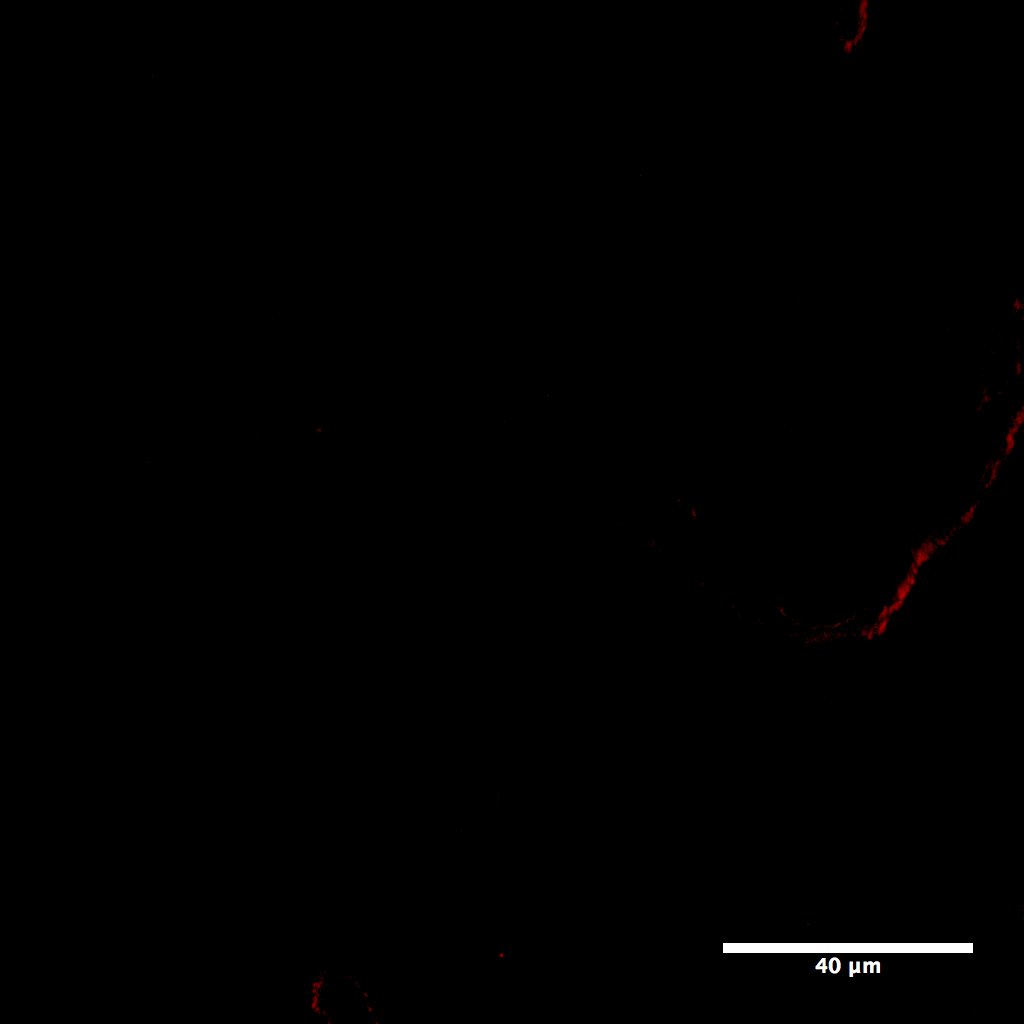
15
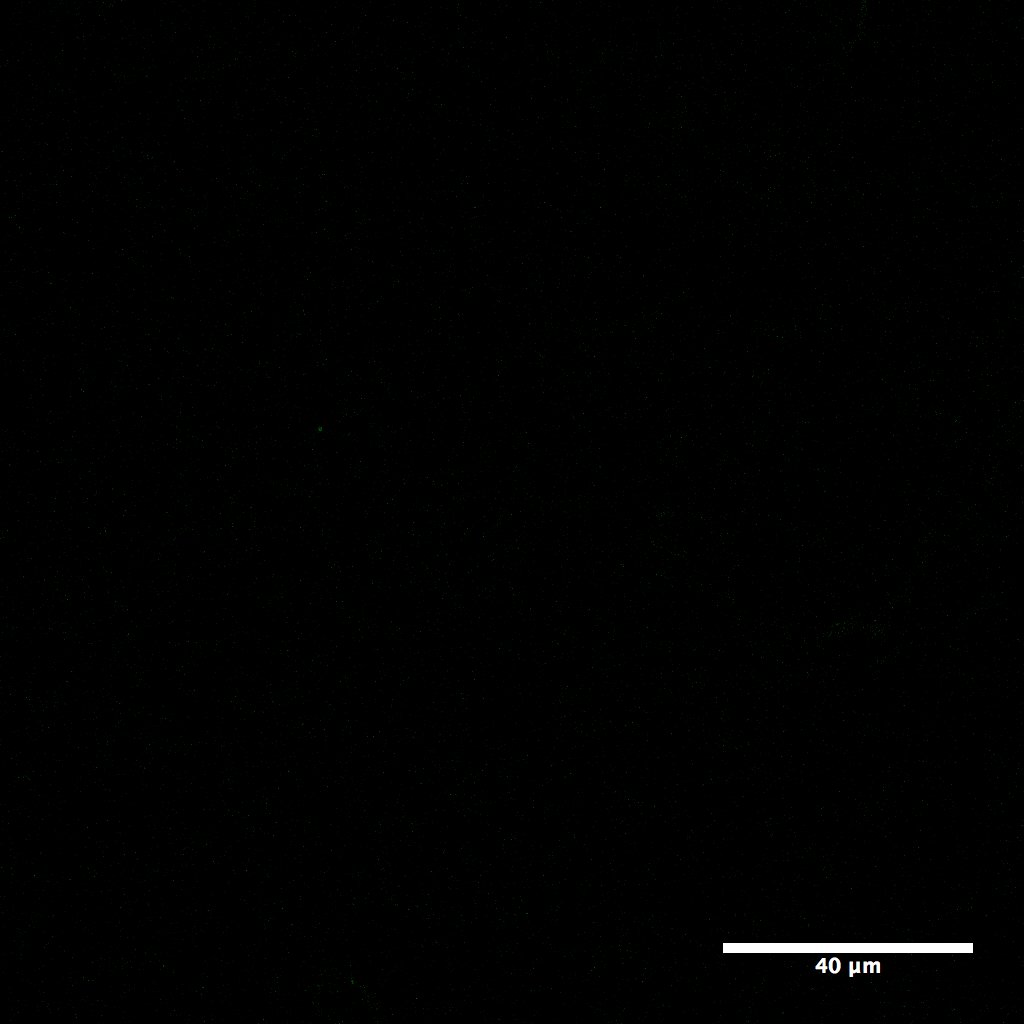
16
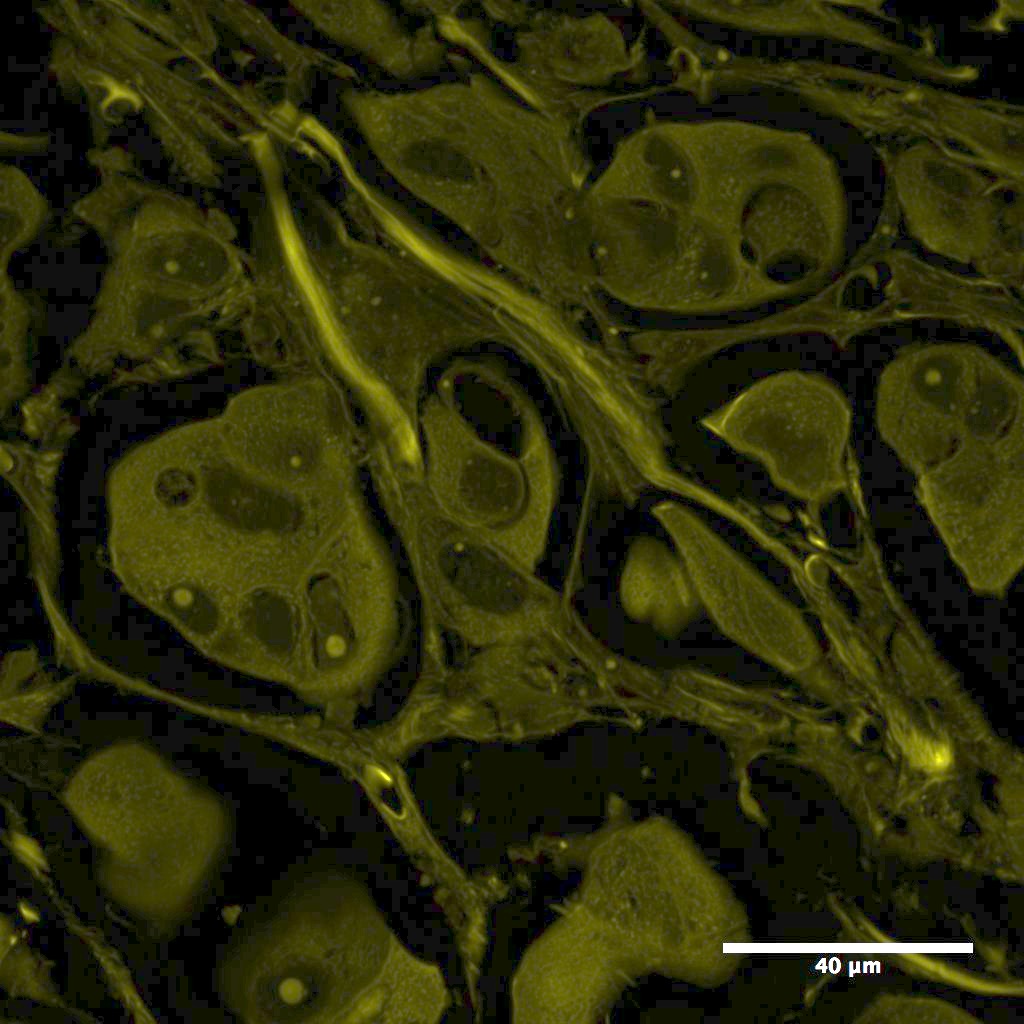
17
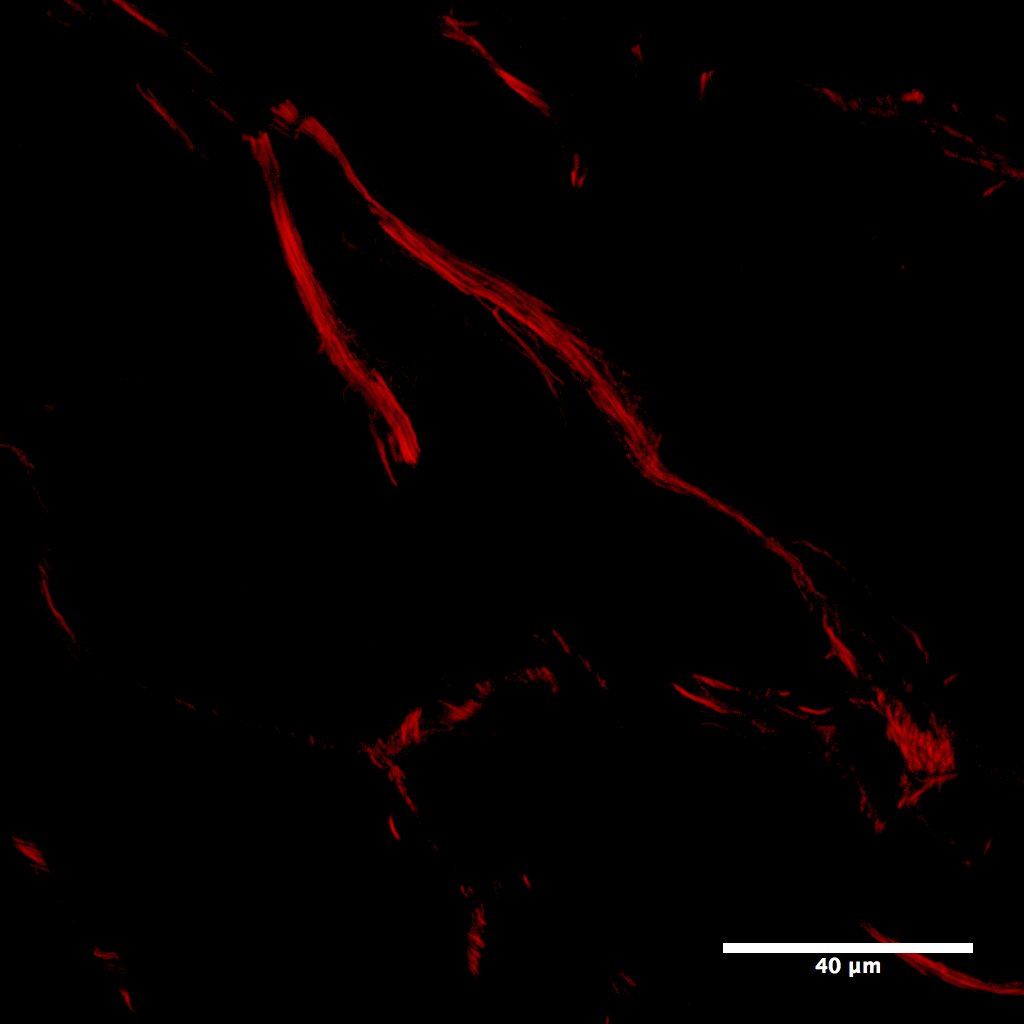
18
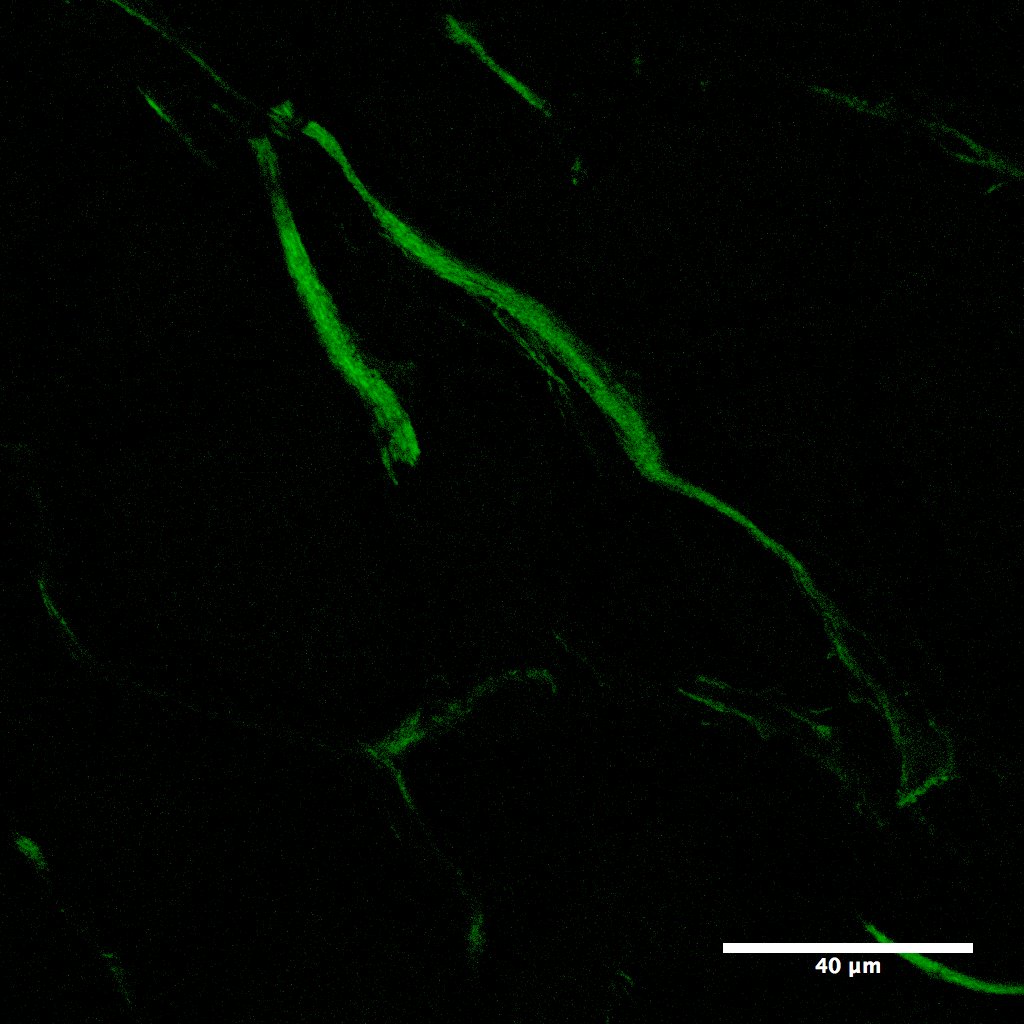


19
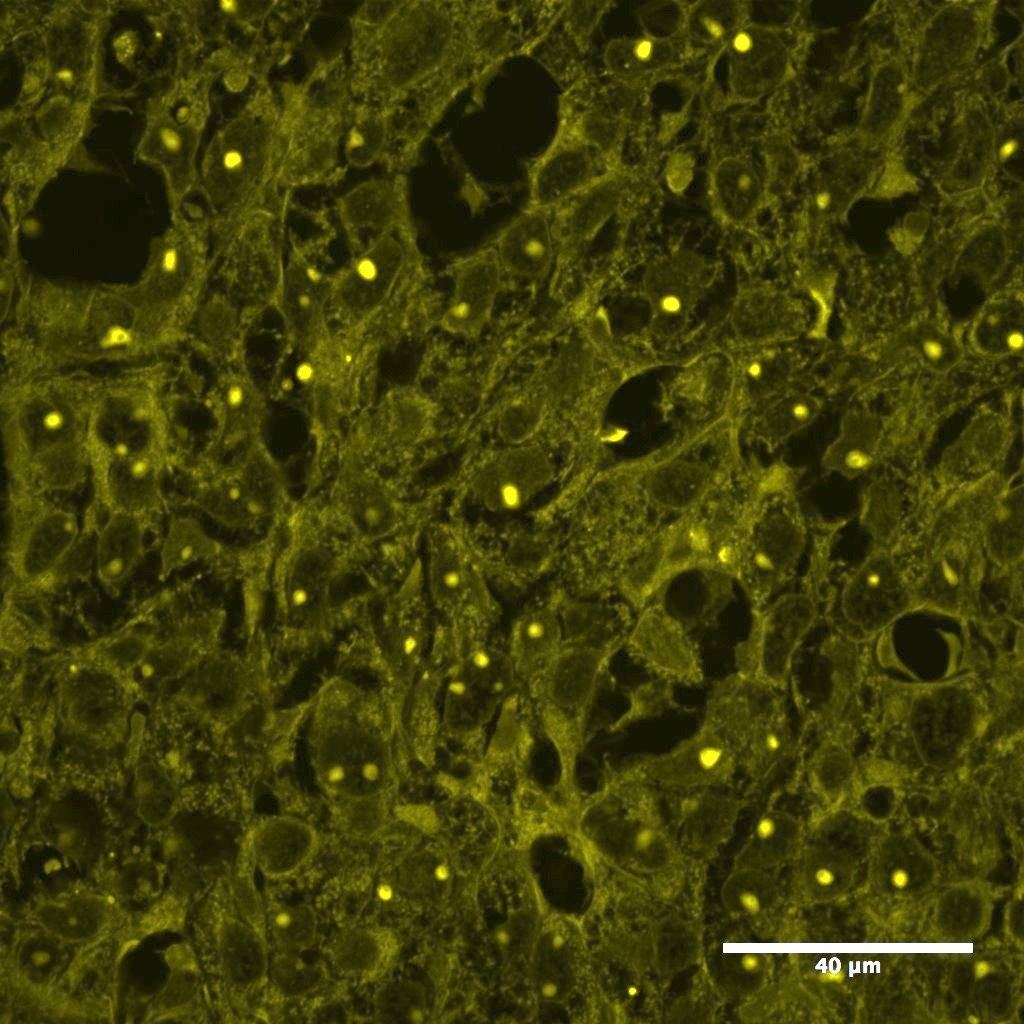
20
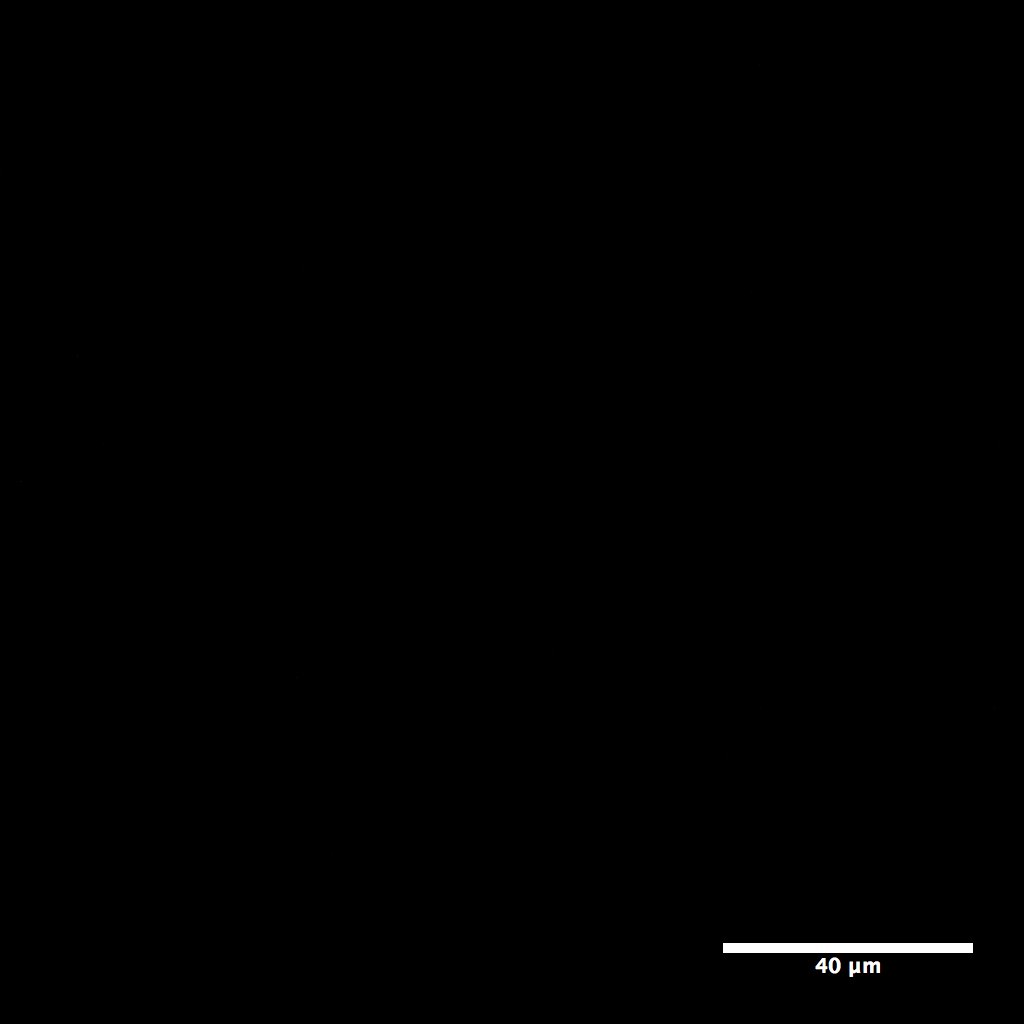
21
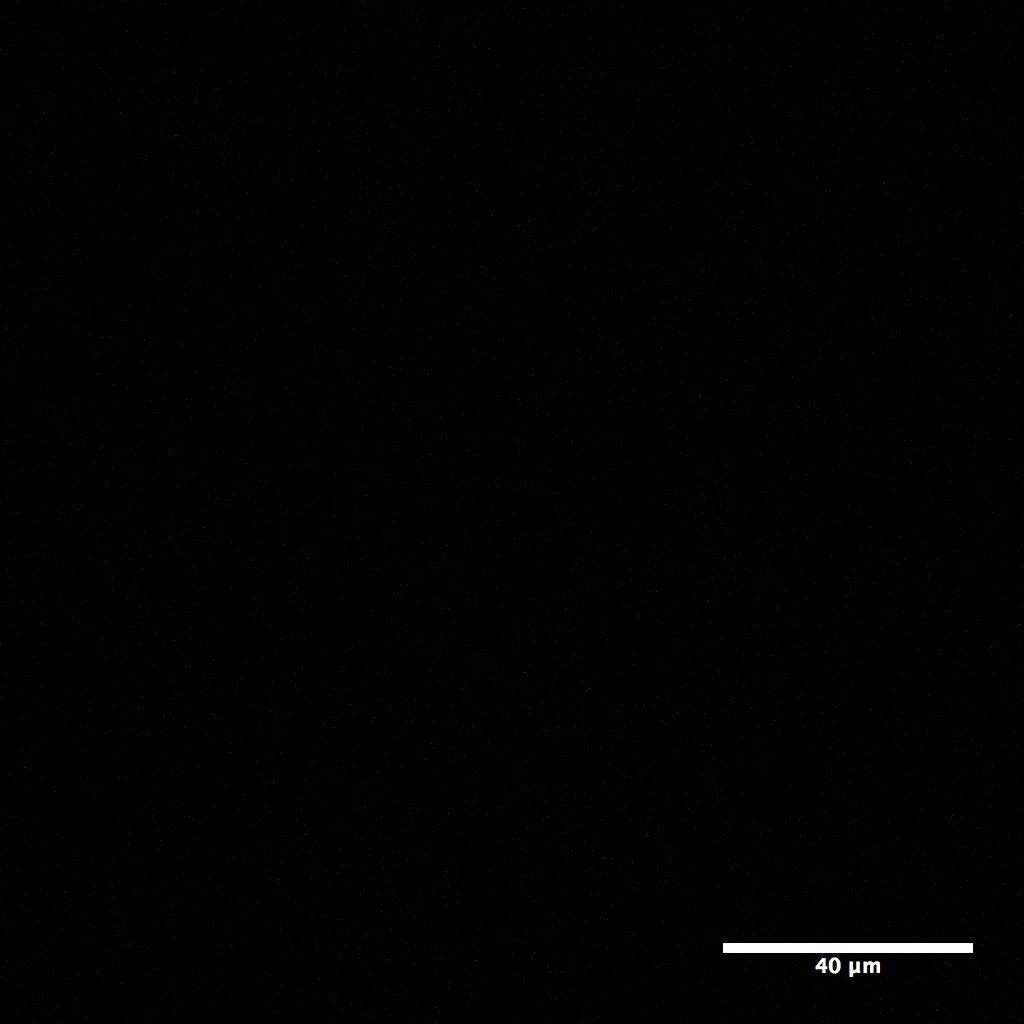
22
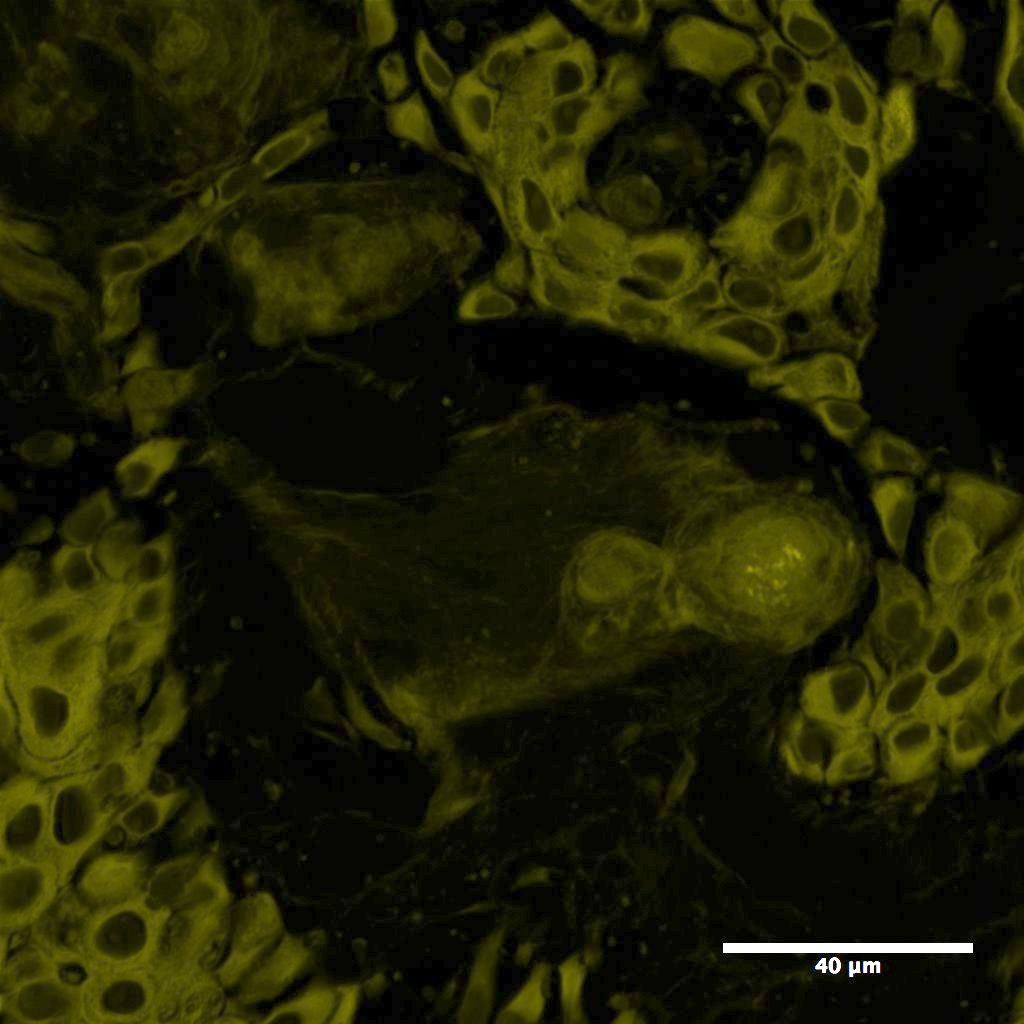
23
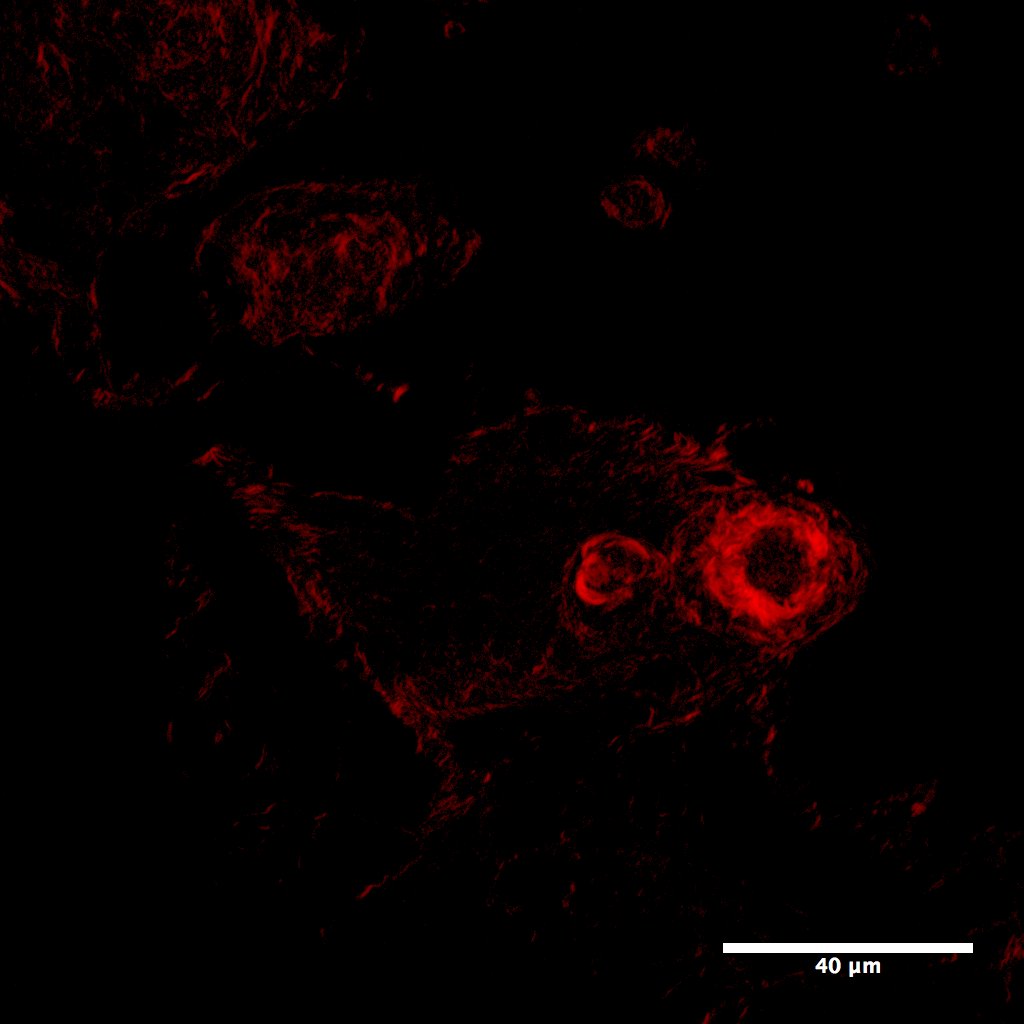
24
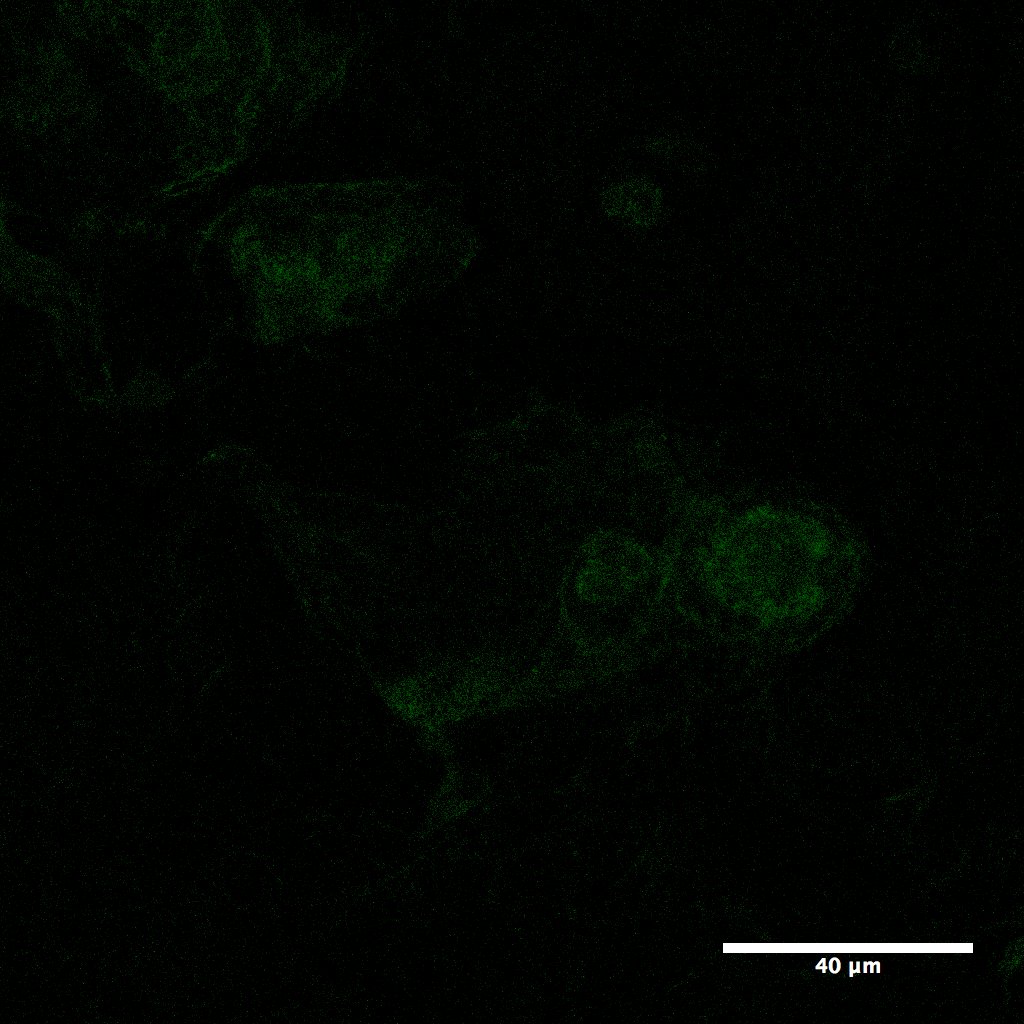


25
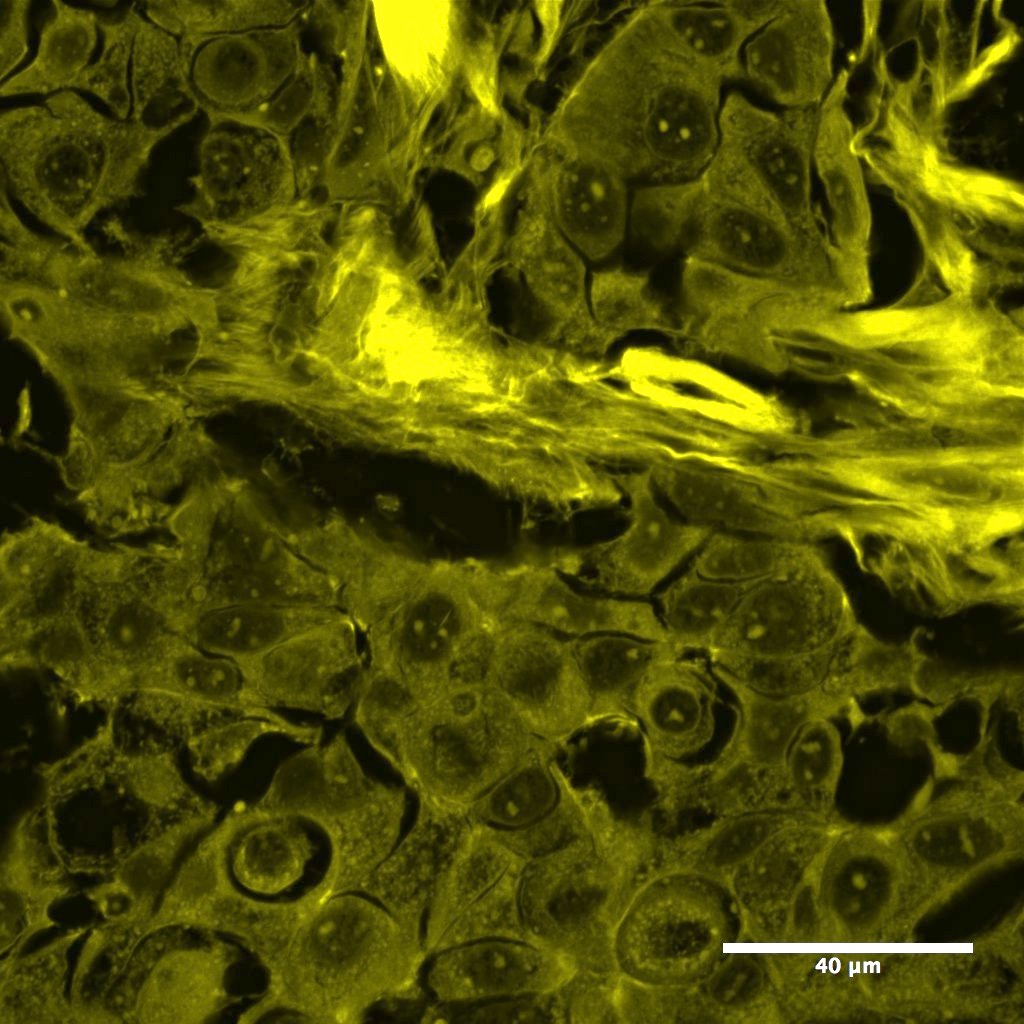
26
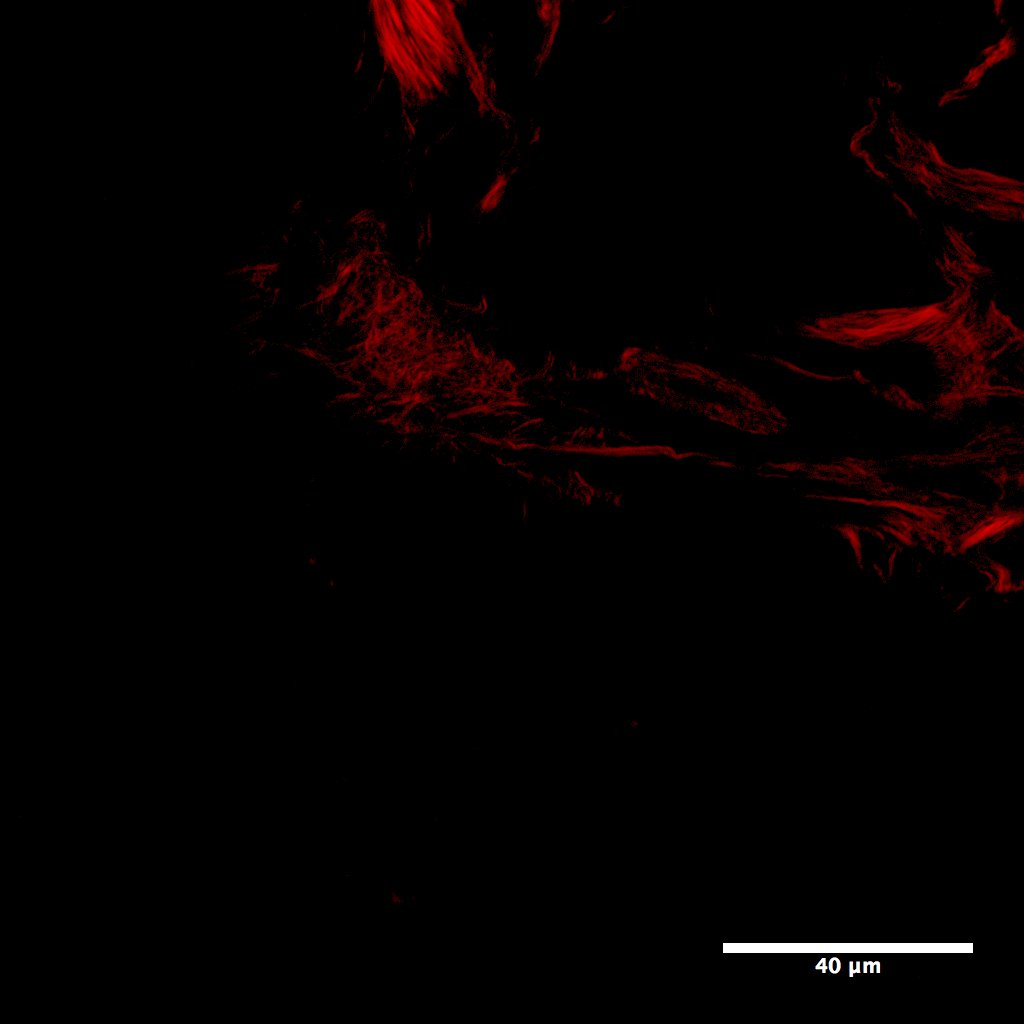
27
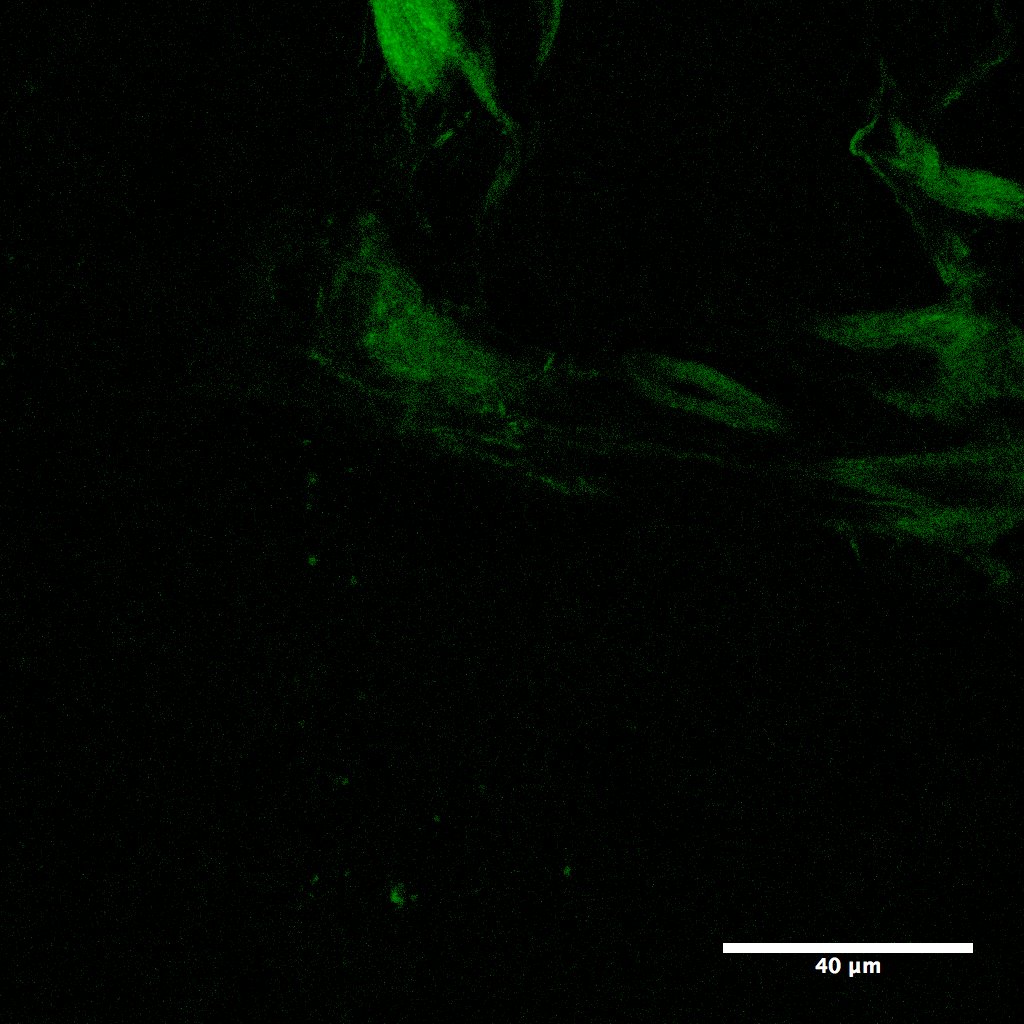


**Supplementary figure 7**: Autofluorescence (01, 04, 07, 10, 13, 16, 19, 22 and 25), fSHG collagen fibers (02, 05, 08, 11, 14, 17, 20, 23 and 26) and bSHG collagen fibers (03, 06, 09, 12, 15, 18, 21, 24 and 27). Images correspond to: invasive ductal carcinoma no special type (01, 02 and 03), classic invasive lobular (04, 05 and 06), tubular (07, 08 and 09), mucinous (10, 11 and 12), papillary (13, 14 and 15), micropapillary (16, 17 and 18), medullary (19, 20 and 21), metaplasic producing-matrix (22, 23 and 24), and invasive apocrine (25, 26 and 27). The images were log-transformed.

***Supplementary references:***

1. Campagnola, P. J. & Loew, L. M. Second-harmonic imaging microscopy for visualizing biomolecular arrays in cells, tissues and organisms. *Nat. Biotechnol.* **21,** 1356–60 (2003).

2. Burke, K., Tang, P. & Brown, E. Second harmonic generation reveals matrix alterations during breast tumor progression. *J. Biomed. Opt.* **18,** 31106 (2013).

3. Coates, A. S. *et al.* Tailoring therapies--improving the management of early breast cancer: St Gallen International Expert Consensus on the Primary Therapy of Early Breast Cancer 2015. *Ann. Oncol.* **26,** 1533–46 (2015).

4. Allred, D. C. *et al.* Adjuvant tamoxifen reduces subsequent breast cancer in women with estrogen receptor-positive ductal carcinoma in situ: a study based on NSABP protocol B-24. *J. Clin. Oncol.* **30,** 1268–73 (2012).

5. Chia, Y. H., Ellis, M. J. & Ma, C. X. Neoadjuvant endocrine therapy in primary breast cancer: indications and use as a research tool. *Br. J. Cancer* **103,** 759–764 (2010).

6. Dellapasqua, S. *et al.* Immunohistochemically Defined Subtypes and Outcome of Apocrine Breast Cancer. *Clin. Breast Cancer* **13,** 95–102 (2013).

7. Houle, M.-A. *et al.* Analysis of forward and backward Second Harmonic Generation images to probe the nanoscale structure of collagen within bone and cartilage. *J. Biophotonics* **8,** 993–1001 (2015).

8. Williams, R. M., Zipfel, W. R. & Webb, W. W. Interpreting second-harmonic generation images of collagen I fibrils. *Biophys. J.* **88,** 1377–86 (2005).

9. Rezakhaniha, R. *et al.* Experimental investigation of collagen waviness and orientation in the arterial adventitia using confocal laser scanning microscopy. *Biomech. Model. Mechanobiol.* **11,** 461–73 (2012).

10. Chen, X., Nadiarynkh, O., Plotnikov, S. & Campagnola, P. J. Second harmonic generation microscopy for quantitative analysis of collagen fibrillar structure. *Nat. Protoc.* **7,** 654–69 (2012).

11. Cox, G. *et al.* 3-dimensional imaging of collagen using second harmonic generation. *J. Struct. Biol.* **141,** 53–62 (2003).

12. Campbell, K. R. & Campagnola, P. J. Wavelength-Dependent Second Harmonic Generation Circular Dichroism for Differentiation of Col I and Col III Isoforms in Stromal Models of Ovarian Cancer Based on Intrinsic Chirality Differences. *J. Phys. Chem. B* **121,** 1749–1757 (2017).

13. Kusafuka, K. *et al.* Cartilaginous features in matrix-producing carcinoma of the breast: four cases report with histochemical and immunohistochemical analysis of matrix molecules. *Mod. Pathol.* **21,** 1282–92 (2008).

14. Psilodimitrakopoulos, S. *et al.* Quantitative discrimination between endogenous SHG sources in mammalian tissue, based on their polarization response. *Opt. Express* **17,** 10168–76 (2009).

15. Campagnola, P. J. *et al.* Three-dimensional high-resolution second-harmonic generation imaging of endogenous structural proteins in biological tissues. *Biophys. J.* **82,** 493–508 (2002).

16. Hammond, M. E. H. *et al.* American Society of Clinical Oncology/College Of American Pathologists guideline recommendations for immunohistochemical testing of estrogen and progesterone receptors in breast cancer. *J. Clin. Oncol.* **28,** 2784–95 (2010).

17. Milanezi, F., Carvalho, S. & Schmitt, F. C. EGFR/HER2 in breast cancer: a biological approach for molecular diagnosis and therapy. *Expert Rev. Mol. Diagn.* **8,** 417–34 (2008).

18. Reis-Filho, J. S. *et al.* Novel and classic myoepithelial/stem cell markers in metaplastic carcinomas of the breast. *Appl. Immunohistochem. Mol. Morphol.* **11,** 1–8 (2003).

19. Cheang, M. C. U. *et al.* Ki67 index, HER2 status, and prognosis of patients with luminal B breast cancer. *J. Natl. Cancer Inst.* **101,** 736–50 (2009).
